# Myocytic androgen receptor overexpression does not affect sex differences in adaptation to chronic endurance exercise

**DOI:** 10.1186/s13293-022-00471-x

**Published:** 2022-10-23

**Authors:** Sabrina Tzivia Barsky, Douglas Ashley Monks

**Affiliations:** 1 Department of Cell & Systems Biology, Faculty of Arts & Science, University of Toronto, Toronto, ON M5S 3G5 Canada; 2 Department of Psychology, University of Toronto Mississauga, 3359 Mississauga Road, Mississauga, ON L5L 1C6 Canada

**Keywords:** Endurance exercise, Sex differences, Muscle androgen receptor, Body composition

## Abstract

Muscle-specific androgen receptor (AR) overexpression (HSAAR transgene) in sedentary male rats results in reduced adiposity, increased mitochondrial enzyme activity, and selective increase in Type 2b myofiber size. Here, we tested chronic endurance exercise interactions with this phenotype in both sexes. Across 9 weeks, rats ran 5×/week on motorized running wheels at increasing speeds and durations. Exercise reduced fat mass in all groups, but sex affected endurance exercise outcomes such that absolute lean mass increased only in females and total body mass decreased only in males. Expected sex differences were observed with males exhibiting greater total body and lean mass; absolute and relative fat mass; bone mineral density; extensor digitorum longus (EDL) myofiber size and glycolytic proportion; but lesser Type 2a and Type 1 myosin expression in tibialis anterior. Observed HSAAR outcomes were not altered by sex, with transgenic rats having greater lean mass, Type 2a myosin expression in soleus, and glycolytic myofiber size in EDL. Tibialis AR content was independently affected by sex, HSAAR, and exercise. No sex differences were observed in tibialis AR expression in wild-type rats, although HSAAR males had greater AR content than HSAAR females. We identified a moderate correlation between AR expression and glycolytic myofiber size, but not whole-body composition. Overall, results suggest myocytic AR overexpression and chronic exercise, despite sharing a similar phenotype to adaptation, are mediated by distinct mechanisms. Further, this study illustrates sex differences in adaptation to chronic endurance exercise, and suggests sex-similarity in the relationship between muscle AR and exercise response.

**Supplementary Information:**

The online version contains supplementary material available at 10.1186/s13293-022-00471-x.

## Highlights

1. Adaptations in bone, lean, and total mass after forced endurance exercise are sex-dependent in rats.
2. Sex differences in muscle fiber-type size and proportion, lean body mass, and bone density are independent of exercise in rats.
3. Myocytic AR overexpression promotes lean body mass and glycolytic myofiber size in both sexes.
4. Skeletal muscle AR protein is elevated by chronic endurance exercise in rats, and these changes in AR content are correlated with improved glycolytic myofiber size.

**Supplementary Information:**

The online version contains supplementary material available at 10.1186/s13293-022-00471-x.

## Introduction

Testosterone promotes sex differences in body composition partly by muscle anabolism and adipose catabolism [1–5]. Treatments that increase circulating androgen levels within hypogonadal males result in losses in fat mass, increases in muscle mass, and improved athletic performance [6–9]. Similar trends in body composition can be seen after physiologically relevant doses of testosterone are re-administered to castrated rats [10–13]. Androgenic effects on body composition in females remain under-studied, and as such, the role of testosterone in female muscle anabolism and athletic performance is less understood [5]. In sports, there is a moderate-to-strong correlation between endogenous circulating androgens and athletic performance in various track and field events in both males and females [14]. Although some studies report additive effects of chronic exercise and combined androgen treatment on muscle phenotype and function in healthy men [15, 16], others do not [17–19].

Muscles vary in their responsiveness to androgens, with strong sexual dimorphism and androgen dependence in the case of perineal muscles associated with the genitals, and a more modest androgen sensitivity and morphological sex difference in other muscles [20–23]. The degree of androgen sensitivity in skeletal muscle is strongly correlated with androgen receptor (AR) expression [24]. In human studies, young healthy females at rest exhibit significantly lower AR expression in vastus lateralis than males [25], as well as significantly lower levels of circulating total and free testosterone at rest and post-exercise [26]. Loss of AR function mutations have established a necessary role for AR in sexual dimorphism of the bulbocavernosus (BC) and levator ani (LA) perineal muscles in rats [27], and in sex differences in adipocyte, bone, and reproductive tissues in mice [28]. Global loss of AR function mutants in XY individuals, including testicular feminization mutation (Tfm), androgen receptor knock-out (ARKO) and complete androgen insensitivity syndrome (cAIS) patients have a comparable phenotype [29, 30], matching growth rates to wild-type XX individuals in young adulthood then drastic development of obesity in later adulthood [31–34]. However, ARKO models produced inconsistent results in the response of limb skeletal muscle. Studies in male AR-null mice have observed some distinct responses in muscle: deficits [35–39], no change [40–42], or increases [43, 44] in either lean body mass, myofiber cross-sectional area, or muscle-specific gene expression. ARKO studies which included voluntary exercise testing identified a worsening of motor coordination, voluntary movement, or resistance to fatigue in global [36, 37, 41, 43, 45] and tissue-specific [46–50] male AR mutants. These results suggest that AR expression may regulate exercise capacity and overall body composition in males; however, the interaction of sex, altered AR expression, and controlled exercise programs on skeletal muscle and adipose response remains unclear.

Previously, we characterized a myocyte-specific AR-overexpression rodent model (HSAAR) made by ligating cloned human AR cDNA into a human skeletal actin expression cassette [51]. To assess the specificity of HSAAR mRNA expression to skeletal muscle, RT-PCR was performed against skeletal, cardiac, and smooth muscle, alongside adipose and kidney of both wild-type (WT) and transgenic (Tg) rats. HSAAR mRNA expression was majorly detectable in extensor digitorum longus (EDL), and detected only a small increase in the urinary bladder and cardiac tissue of Tg but not WT rats suggestive of some transgene expression in smooth and cardiac muscle in addition to the robust overexpression in skeletal muscle. No HSAAR expression was observed in adipose or kidney of Tg rats, suggesting that transgene expression is restricted to muscle as would be expected from the promoter used. In a 10-week observation of HSAAR body composition by dual X-ray absorptiometry (DXA), Fernando et al. [52] reported increased lean body mass percentage and decreased absolute fat mass in male HSAAR Tg rats compared to WT littermates. These results were reproduced in female HSAAR Tg rats with 4-week testosterone treatment showing the dependence of hormonal circulation on AR-mediated alterations in females. In addition, sedentary HSAAR Tg males exhibit selective increases in Type 2b hypertrophy in EDL, significant reductions in adiposity and adipocyte size, increased resting oxygen consumption, and increased activity of Complex I–IV in the mitochondrial ETC. Other works using pharmacological manipulation of AR highlight its role in modulating mitochondrial activity and muscle adaptation in male and female rats. Inhibition of AR activity using flutamide decreases the activity of key mitochondrial oxidative enzymes in sedentary male rats [53] and inhibits both resistance and endurance exercise-mediated muscle hypertrophy in trained male rats [54]. Selective androgen receptor modulators (SARMs) used in sedentary female rats present a dose-dependent, muscle fiber-specific increase in myofibrillar fractional synthesis rate which was strongly associated with increased muscle mass [55].

Fiber type-specific adaptation, fat loss, and mitochondrial biogenesis are hall-mark effects of chronic endurance exercise and these adaptations to exercise are enhanced when combined with androgenic supplementation in both rats and mice. Fontana et al. [56] reported increased muscle mass in male mice after 6 weeks of high-intensity treadmill running, with an additive effect on muscle weight with mesterolone treatment. Interestingly, muscle fiber type distribution showed limited effects by anabolic steroids alone, but slow-twitch oxidative fiber cross-sectional area (CSA) was increased when endurance exercise was combined with mesterolone administration [56]. Alternatively, a 6-week treadmill protocol found neither endurance exercise nor anabolic steroids altered muscle mass or CSA in male rats [57], but exercise or steroids alone reduced adiposity and was further reduced with the two treatments combined. This points to a complicated relationship on body composition between circulating androgenic activity and exercise, especially when in combination.

There has been increased interest in the role of AR expression levels within muscle due to strong hypotheses of direct anabolic action through chronically activated transcription and translation post-exercise. Morton et al. [58] observed that male participants with higher AR expression in muscle exhibited larger changes in muscle hypertrophy and lean body accretion in a 12-week resistance exercise program, compared to participants with lower AR expression in muscle. Furthermore, these adaptations in muscle were strongly correlated to higher levels of intramuscular AR, but not to circulating testosterone or dihydrotestosterone (DHT), which remained unchanged between high and low exercise-responders and exercise treatment. Tissue-specific genomic AR modification provides an optimal model to explore the impact of supraphysiological intramuscular AR expression on muscle phenotype under controlled and chronic exercise treatments. However, investigation of the causal relationship between intramuscular AR and exercise adaptation are absent, as studies of mouse AR mutants have typically been performed in sedentary animals. In addition, work in both human and rodent literature has typically focused on male populations, and we have a limited understanding of chronic exercise and androgenic responses in females.

The objective of this work is to evaluate sex differences in adaptation to chronic endurance exercise and their interaction with myocytic androgen receptor overexpression. We, therefore, examined whole-body composition, muscle gene expression, and oxidative fiber typing following chronic exercise in both female and male HSAAR.

## Materials and methods

### Animals

All animal handling and experimental procedures were performed at the University of Toronto Mississauga (UTM) Animal Facility and were approved by the Biological Sciences Local Animal Care Committee, complying with guidelines established by the University of Toronto Animal Care Committee and the Canadian Council on Animal Care (Approved protocol #20012103). All experiments were performed using 60- to 90-day-old male (*n* = 46) and female (*n* = 48) HSAAR transgenic (Tg) and wild-type (WT) rats bred on a Sprague Dawley (SD) background. Wild-type rats were purchased from Charles River. All HSAAR and WT rats were bred in-house to generate the appropriate genotypes for this study. All rats were pair-housed under standard environmental conditions (20–22 °C, 12:12 h light–dark cycle), with water and standard rat chow (44.9% carbohydrates, 9% lipids, 19% protein; Envigo Teklad) provided ad libitum.

### Genotyping

**Fig. 1:**
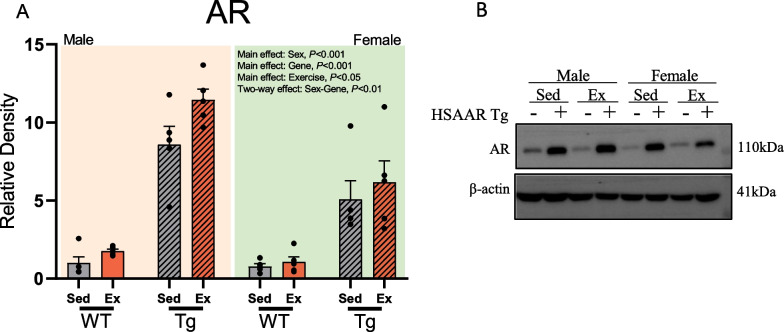
Changes in skeletal muscle androgen receptor protein content are driven by sex differences and chronic aerobic exercise in tibialis anterior. Quantification of western blot analysis of **a** androgen receptor (AR) from tibialis anterior (TA) muscles, and **b** the representative immunoblots between males and females with their respective grouping by exercise and HSAAR Tg genotype. Indicated groups: males (*yellow*), females (*green*), sedentary (*grey*), aerobic exercise (*red*), wild-type (wt) (*blank fill*), and HSAAR transgenic (Tg) (*hatch fill*). Data expressed in relative density (R.D.) and presented as means ± s.e.m. Data analyzed using three-way ANOVA with post hoc Tukey HSD for multiple comparisons. **a** *n* = 5 per sex–genotype–exercise group

**Fig. 2:**
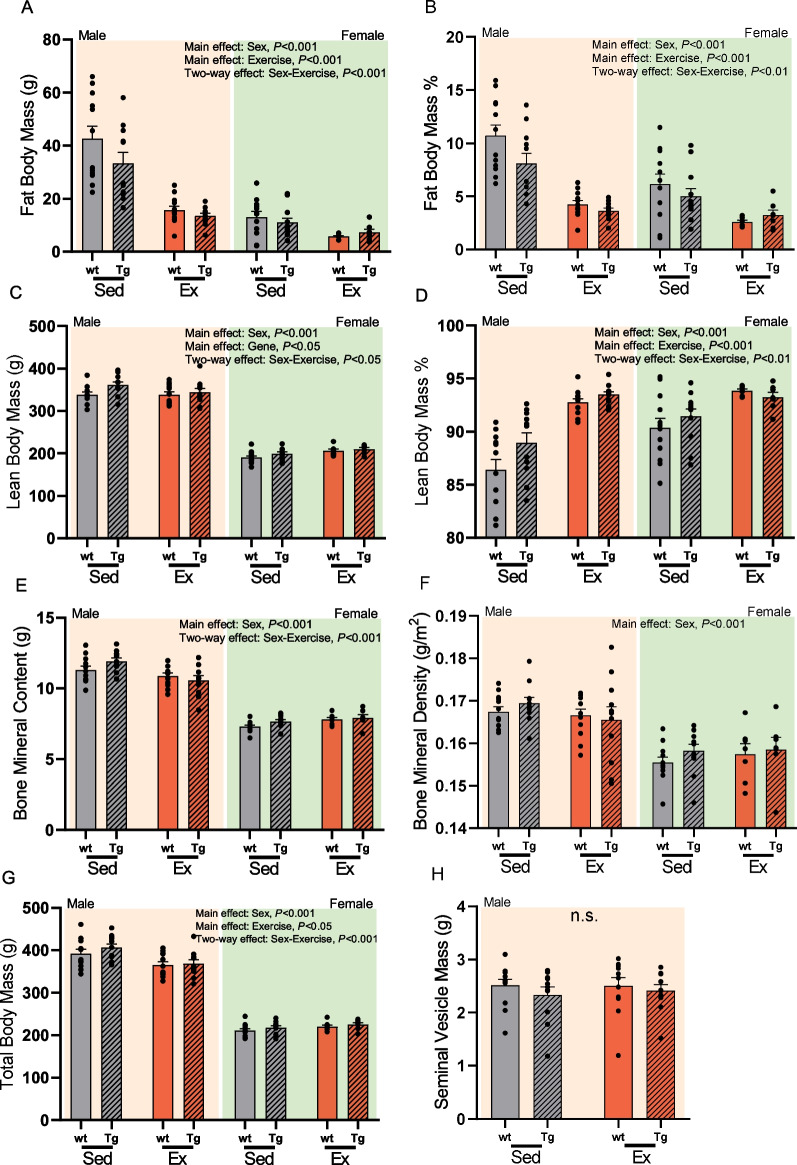
End-point dual-energy X-ray absorptiometry (DXA) measurements show that endurance exercise improves body composition through reduced adiposity, while sex differentially affects post-exercise DXA lean mass, total mass, and bone mineral content. Panels include **a** fat body mass (FBM), **b** fat body mass percent (FBM%), **c** lean body mass (LMB), **d** lean body mass percent (LBM%), **e** bone mineral content (BMC), **f** bone mineral density (BMD), **g** total body mass (TBM) of *N* = 94 Sprague Dawley rats after 9 weeks of aerobic training. Global androgenic activity control (**h**) seminal vesicle mass (SVM) measured at end-point collection in all males. Data from indicated groups: males (*yellow*), females (*green*), sedentary (*grey*), aerobic exercise (*red*), wild-type (wt) (*blank fill*), and HSAAR transgenic (Tg) (*hatch fill*). Data are presented as means ± s.e.m and analyzed using three-way LMER ANOVA with post hoc Bonferroni-corrected *t*-tests for multiple comparisons for (**a**–**g**), and two-way ANOVA with post hoc Tukey HSD for (**h**). **a**–**g** *n* = 7 per female–genotype–exercise groups, *n* = 11–12 per male–genotype–exercise groups. **h** *n* = 11 per genotype–exercise group

All weaned animals used in the endurance exercise paradigm were genotyped for the presence of the HSAAR transgene. HSAAR animals have an observed sevenfold increase in AR protein expression in whole skeletal muscle (Fig. 1A), yet have endogenous functional AR, as shown by the normal mass of dissected seminal vesicles in males (Fig. 2H). Detailed methodology on production, genotyping, and tissue-isolation validity of HSAAR Tg has been previously reported [51]. Animals were ear-notched and DNA was extracted using the “HotSHOT” method [59]. Briefly, animals carrying the HSAAR transgene were identified using PCR amplification with the following primers: (forward) 5ʹ-GGACAGGGCACTACCGAG-3ʹ and (reverse) 5ʹ-GGCTGAATCTTCCACCTAC-3ʹ. Thermal cycler conditions were optimized to 35 cycles of 2 min at 95 °C, 50 s at 95 °C, 1 min at 59.6 °C, 1 min 20 s at 72 °C, with a single and final cycle of 4 min at 72 °C. Amplified HSAAR fragments were run on a 2% agarose gel and Et-Br stained with appropriate HSAAR controls.

### Endurance exercise

Rats at post-natal day 30 (PND-30) were randomly assigned into the following groups: HSAAR male exercise (*n* = 11), HSAAR male sedentary (*n* = 11), WT male exercise (*n* = 12), WT male sedentary (*n* = 12), HSAAR female exercise (*n* = 12), HSAAR female sedentary (*n* = 12), WT female exercise (*n* = 12), and WT female sedentary (*n* = 12). We utilized a 6-wheel motorized running wheel set-up (80805, Lafayette Instrument). Exercise animals were subjected to a running-wheel familiarization period, adapted from Fontana et al. [56], which was followed by a forced endurance protocol, adapted from Smolka et al. [60] (Table 1). Animals who could not successfully complete running sessions after the familiarization period were not included in the final body composition analyses (see “Exclusion criteria”). Sedentary groups were handled to match that within exercise groups. Exercise began between 8:00 AM–10:00 AM. All cohorts began exercise at the age of PND60–90.

**Table 1:** Endurance running paradigm, adapted from [60] (5 days/week), using motorized running wheel set-up (80805, Lafayette Instrument)

Week	Speed (m/min)	Duration (min)
Familiarization	10	15
1	15	20
2	20	30
3	20	45
4–9	22	60

### Exclusion criteria

Although exercise behavior was not quantified, there was a noticeable difference in the style of exercise performance of a subset of female rats, which presented as isometric holding of the running wheel rods, followed by a sprint towards the front of the running wheel. Rats with atypical running behavior were classified as “non-conforming” and excluded from analyses due to evidence presented hereafter that this atypical style did not result in expected changes in body composition (*n* = 5, WT female; *n* = 5, HSAAR Tg female). Subjects were also excluded if they were otherwise unable to complete the exercise program due to injury (*n* = 1, HSAAR Tg male).

### Whole-body composition

Immediately following the final bout of exercise, both exercise and sedentary cohorts underwent sedation using inhalant anesthesia with 4–5% isoflurane dispensed at 1–2 L/min. We used dual-energy X-ray absorptiometry (DXA) scanning (QDR 4500; Hologic) and its respective rat whole-body software (Hologic QDR Software, Version 12.3) to measure total body mass (TBM), fat body mass (FBM), fat body mass percent (FBM%), lean body mass (LBM), lean body mass percent (LBM%), bone mineral content (BMC), and bone mineral density (BMD) (*N* = 94).

### Dissection

All dissections were performed 24 h following the final bout of exercise. Animals were anesthetized by intraperitoneal injection of 5% avertin (0.05 g/mL tribromoethanol, 300 mg/kg). Gastrocnemius (GAST), soleus (SOL), tibialis anterior (TA), and extensor digitorum longus (EDL) were harvested. Seminal vesicles were dissected and weighed to confirm androgen status in males. SOL and EDL were trimmed, set in OCT embedding medium, and fresh frozen in liquid nitrogen. GAST and TA were fresh frozen on dry ice. Blood samples were spun at 4000×*g* at 4 °C for 20 min, and plasma was collected. All tissues were stored at − 80 °C until further processing.

### Histology and image acquisition

Serial transverse sections of EDL and SOL were cut at − 23 °C (12 µm) (CM3050S, Leica Biosystems). All slices were mounted on SuperFrost Plus microscope slides (ER4951PLUS, Fisherbrand), air-dried, and stored at − 80 °C until subsequent staining. Oxidative and glycolytic myofiber type was identified using succinate dehydrogenase (SDH) staining and overlayed to H&E-stained subject-matched sections to quantify oxidative and glycolytic fiber distribution, proportion, and size (*n* = 6 per group). Minor ellipse length was used as an indirect measure of cross-sectional area to provide more robust protection against slicing angle artifact on area measures [52]. Images were acquired using an Olympus bright-field microscope (model BX51; Olympus, Tokyo, Japan), a ×4 and ×40 objective, equipped with an Olympus Digital Camera for bright-field pictures. Images were chosen based on a centered field of view across the cryo-sectioned muscle, and fibers were sampled about that point. Two images were taken per rat (*n* = 6 per group); one image across SDH-stained EDL and another myofiber-matched overlayed image across H&E-stained EDL. At ×40 objective, the average count of fibers measured was 58 ± 3 and 36 ± 2 in female and male EDL, respectively. Identical microscope settings were used for imaging samples within each experiment. Photomicrographs were imported into ImageJ software version 1.53c (National Institutes of Health, Bethesda, MD), which was used to trace cell size. All imported photomicrographs were given randomized identification labels to blind the experimenter to independent group factors.

### Hematoxylin and eosin, H&E

Slides were thawed to room temperature (1 h) and fixed in 4% paraformaldehyde in 0.01 M PBS for 15 min, then rinsed in several washes of distilled water, and stained with Harris Hematoxylin (HHS32, Sigma-Aldrich) for 15 min. Sections underwent differentiation in 1% acid–alcohol, a quick bluing step in 0.2% ammonia water, and several water rinses. Slides were counter-stained with eosin Y (EOS109, BioShop). Sections were dehydrated by a series of graded ethanol, cleared by xylene, and cover-slipped using a solvent-based mounting medium (Cytoseal 60™, Thermo Scientific).

### Succinate dehydrogenase, SDH

Slides were thawed to room temperature for 20 min, and incubated in 0.05% nitrotetrazolium blue chloride (N6876, Sigma-Aldrich), 0.1 M sodium succinate (S2378, Sigma-Aldrich), and 0.01 M PBS at 37 °C for 40 min with gentle rocking. Sections were dehydrated in a series of graded ethanols, cleared by xylene, and cover-slipped by a solvent-based mounting medium (8310-16, Thermo Scientific).

### Protein preparation

Dissected TA and SOL (120–150 mg) were suspended in 700 uL of radioimmunoprecipitation assay (RIPA) buffer (50 mM Tris–HCl pH 8.0, 2 mM EDTA, 1% NP-40, 0.5% sodium deoxycholate, 0.1% SDS, 150 mM NaCl) supplemented with protease inhibitor cocktail (P8340, Sigma 1:500). The muscles were manually minced and incubated on ice for 20 min, shaking every 5 min. Whole muscle lysates were centrifuged at 14,000×g for 20 min at 4 °C. The supernatant was extracted, and protein concentration was determined using the Bradford method and bovine serum albumin standards. Aliquoted whole-muscle lysates were diluted in 6×SDS loading buffer (60% glycerol, 375 mM Tris–HCl pH 6.8, 12% SDS, 0.12% bromophenol blue) and 1 M DTT (161-0610, Bio-Rad, 1:20) before being stored at − 80 °C.

### Western blotting

Lysates were boiled at 70 °C for 10 min and equal amounts of protein were separated on 7% SDS-PAGE gel for MHC1, MHC2a, PGC1α, and AR, or 10% SDS-PAGE for TFAM and NRF-2. SDS-PAGE was run at 80 V for 30 min and 100 V for 60 min to resolve protein bands, followed by wet transfer using 10% methanol and 0.1% SDS onto a PVDF membrane (Immobilon-P, EMD Millipore) at 17 V for 24 h (EI9051, Invitrogen). Membranes were rinsed in TBS 0.1% Tween-20 (TBS-T) (5 min) and blocked with 5% skim milk in TBS-T (60 min) at room temperature. Primary antibodies were diluted with 3% skim milk in TBS-T and incubated overnight at 4 °C. Washes were performed with TBS-T (3 × 5 min) followed by a room temperature incubation in secondary antibody diluted with 3% skim milk in TBS-T (60 min). Washes were performed with TBS-T (3 × 5 min, followed by 10 min) before the membrane was incubated for 2 min with ECL (WBLUF0100, Immobilon Forte Western HRP Substrate, Millipore). Next, digital chemiluminescent imaging was completed on the ImageQuant LAS 500 (GE Healthcare Life Sciences) with 30- to 60-s exposure. Equal protein loading across samples was demonstrated by beta-actin expression, and experimental bands were normalized to sample-matched actin intensity. Loading controls were run on the same blot. Immunoblots were visualized using ImageJ software version 1.53c.

### Antibodies

For immunoblotting, antibodies used in this study were purchased from EMD Millipore (Oakville, Canada), Sigma Aldrich (Oakville, Canada), Proteintech (Illinois, USA), and Cell Signaling Technology (Whitby, Ontario). The following primary antibody was used from EMD Millipore (Oakville, Canada): anti-androgen receptor (Millipore Cat# 06-680, 1:1,000). The following primary antibodies were used from Sigma Aldrich (Oakville, Canada): anti-fast skeletal myosin (M4267, 1:15,000), anti-slow skeletal myosin (Sigma-Aldrich Cat# M8421, 1:10,000), and anti-beta actin (Sigma-Aldrich Cat# A2066, 1:10,000). The following primary antibodies were used from Proteintech (Illinois, USA): anti-PGC1α (Proteintech Cat# 66369-1-Ig, 1:5,000), anti-NRF-2 (Proteintech Cat# 16396-1-AP, 1:500), and anti-TFAM (Proteintech Cat# 22586-1-AP, 1:1,000). The following HRP-conjugated secondary antibodies were used from Cell Signaling Technology (Whitby, Ontario): goat anti-rabbit IgG, HRP-linked (Cell Signaling Technology Cat# 7074, 1:5,000) and horse anti-mouse IgG, HRP-linked (Cell Signaling Technology Cat# 7076, 1:5,000).

### Statistics

All analyses were completed using R (Version 4.0.2). For whole-body DXA measurements, main and interactive effects by sex, HSAAR genotype, and endurance exercise were analyzed using a linear mixed-effects model (LMER, *lmer* function of the *lmeTest* package) which is used on normal data with heteroskedasticity [61]. When indicated by the Satterthwaite’s DF *p*-value in LMER, a Bonferroni-corrected *t*-test was used for post hoc analysis for multiple comparisons of interactive effects. For immunoblotting and histology, normality and variance were verified using Shapiro–Wilk’s and Bartlett’s tests, respectively. For myosin protein isoforms, mitochondrial biomarkers, myofiber minor ellipse, and myofiber proportions, the main and interactive effects of sex, HSAAR genotype, and endurance exercise were analyzed by three-way ANOVA and post hoc Tukey analysis for multiple comparisons. All data are presented as mean ± standard error of the mean (S.E.M) with statistical significance set at *α* = 0.05.

## Results

### Body composition following chronic exercise in atypically running females

Ninety-five Sprague Dawley rats were bred to perform this experiment, but only 94 rats (Males: *n* = 46, Females: *n* = 48) completed the exercise paradigm due to injury-related attrition of one male mid-experiment. Twenty-four female rats were subjected to forced treadmill running, but those rats with non-conforming exercise performance were tracked, and end-point 9-week DXA measurements were compared between conforming (CF) and non-conforming (nCF) exercising females (nCF: *N* = 10; nCF-Tg: *n* = 5, nCF-WT: *n* = 5) to identify any effects of running style on body composition. The results of the non-paired, two-sided *t*-tests highlighted the significant differences in absolute lean mass (*P* < 0.001), total body mass (*P* < 0.001), bone mineral content (*P* < 0.01), and bone mineral density (*P* < 0.05) across CF and nCF exercising females (Additional file 1: Table S1). Only data from female rats with correct exercise technique were kept (i.e., DXA, immunoblots, and histology) (F-Ex-Tg: *n* = 7, F-Ex-WT: *n* = 7). All males recruited to the exercise group exhibited appropriate forced running-wheel exercise techniques (M-Ex-Tg: *n* = 11, M-Ex-WT: *n* = 12). Sedentary rats were not subjected to any performance-based exclusion criteria (F-Sed-Tg: *n* = 12, F-Sed-WT: *n* = 12, M-Sed-Tg: *n* = 11, M-Sed-WT: *n* = 12).

### Tibialis anterior androgen receptor expression only exhibits sexual dimorphism between HSAAR transgenic rats, but not between wild-type males and females

Using tibialis anterior, we validated the presence of overexpression of AR in HSAAR compared to wild-type subjects. A three-way ANOVA revealed additional main effects of sex and main effects of exercise on AR expression. Compared to sedentary controls, all exercised rats exhibited increased AR expression in dissected TA skeletal muscle (Fig. 1A; Table 2). Sex presented as a main effect where males had significantly higher levels of myocytic AR than their female counterparts (Fig. 1A; Table 2). There was an interactive effect of sex and HSAAR Tg on AR expression, which appears to be driven by sex differences in the transgenic groups but not in the wild-type groups, such that transgenic males had higher AR expression than transgenic females, but no sex difference was observed in wild-types (Fig. 1A; Table 2). Altogether, 9 weeks of forced endurance exercise increase the protein expression of AR in glycolytic limb muscle at rest. Additionally, sex differences in skeletal muscle AR expression exist only under the influence of the HSAAR transgene, but not wild-type conditions, where transgenic males have almost twofold greater AR presence than transgenic females.

**Table 2:** Sex, HSAAR genotype, and endurance exercise effects on tibialis anterior androgen receptor (AR) protein expression

	Effect	*F* statistic (*df*)	Groups		*T* statistic	*P*-value
			Mean ± SEM	Mean ± SEM		
AR (R.D.)	Sex	*F*(1,37) = 17.36	F: 3.28 ± 0.69	M: 5.71 ± 1.07	–	< 0.001
	Gene	*F*(1,37) = 131.44	Tg: 7.83 ± 0.76	WT: 1.16 ± 0.15	–	< 0.001
	Exercise	*F*(1,37) = 4.68	Ex: 5.12 ± 1.02	Sed: 3.86 ± 0.84	–	< 0.05
	Sex × gene	*F*(1,37) = 11.45			–	< 0.01
			M-Tg: 10.03 ± 0.79	F-Tg: 5.63 ± 0.88	3.716	< 0.001
			F-WT: 0.93 ± 0.18	F-Tg: 5.63 ± 0.88	− 5.262	< 0.001
			M-WT: 1.39 ± 0.24	F-Tg: 5.63 ± 0.88	− 4.684	< 0.001
			F-WT: 0.93 ± 0.18	M-Tg: 10.03 ± 0.79	− 11.171	< 0.001
			M-WT: 1.39 ± 0.24	M-Tg: 10.03 ± 0.79	− 10.428	< 0.001
			M-WT: 1.39 ± 0.24	F-WT: 0.93 ± 0.18	1.543	n.s

Data expressed as relative density (R.D.). F, female (*n* = 20); M, male (*n* = 20); Ex, exercise (*n* = 20); Sed, sedentary (*n* = 20); Tg, HSAAR transgenic (*n* = 20); WT, wild-type (*n* = 20). Data analyzed using three-way ANOVA with post hoc Tukey HSD for multiple comparisons. Non-significant interactive effects not shown, see Additional file 1: Tables S4 and S5

### Sex affected all DXA-measured body composition parameters except for relative lean mass

End-point DXA measurements revealed significant differences in male and female body composition. Compared to the female subjects, males had significantly higher absolute fat mass (FBM), fat mass % (FBM%), absolute lean mass (LBM), total body mass (TBM), bone mineral content (BMC), and bone mineral density (BMD) (Fig. 2A–G; Table 3). However, lean body mass percent (LBM%) was higher in females compared to males.

**Table 3:** Summarized main effects of sex, HSAAR genotype, or endurance training on whole-body DXA measurements

	Effect	*F* statistic (*df*)	Groups		*P*-value
			Mean ± SEM	Mean ± SEM	
Fat body mass (g)	Sex	*F*(1,81) = 73.87	F: 10.02 ± 0.95	M: 26.41 ± 2.42	< 0.001
	Exercise	*F*(1,81) = 54.84	Ex: 11.55 ± 0.90	Sed: 24.85 ± 2.57	< 0.001
Fat body mass %	Sex	*F*(1,81) = 21.09	F: 4.60 ± 0.43	M: 6.72 ± 0.56	< 0.001
	Exercise	*F*(1,81) = 59.14	Ex: 3.56 ± 0.19	Sed: 7.49 ± 0.54	< 0.001
Lean body mass (g)	Sex	*F*(1,81) = 1020.47	F: 199.68 ± 2.35	M: 345.38 ± 3.77	< 0.001
	Gene	*F*(1,81) = 5.51	Tg: 283.66 ± 12.27	WT: 275.46 ± 11.30	< 0.05
Lean body mass %	Sex	*F*(1,81) = 12.52	F: 91.89 ± 0.42	M: 90.37 ± 0.56	< 0.001
	Exercise	*F*(1,81) = 61.01	Ex: 93.29 ± 0.17	Sed: 89.30 ± 0.51	< 0.001
Total body mass (g)	Sex	*F*(1,81) = 918.20	F: 217.32 ± 2.38	M: 382.94 ± 4.99	< 0.001
	Exercise	*F*(1,81) = 5.26	Ex: 312.11 ± 12.27	Sed: 304.80 ± 14.08	< 0.05
Bone mineral content (g)	Sex	*F*(1,81) = 481.90	F: 7.63 ± 0.08	M: 11.17 ± 0.15	< 0.001
Bone mineral density (g/m^2^)	Sex	*F*(1,81) = 52.76	F: 0.157 ± 0.001	M: 0.167 ± 0.001	< 0.001

F, female (*n* = 38); M, male (*n* = 46); Ex, exercise (*n* = 37); Sed, sedentary (*n* = 47); Tg, HSAAR transgenic (*n* = 41); WT, wild-type (*n* = 43). Summarized data presented here were analyzed using three-way LMER ANOVA. Non-significant main effects not shown, see Additional file 1: Table S2

### HSAAR overexpression increased lean body mass regardless of sex

Across all subjects, LBM was significantly increased in HSAAR expressing rats compared to their wild-type counterparts (Fig. 2C; Table 3). Myocytic AR overexpression did not significantly affect FBM, FBM%, LBM%, TBM, BMC, or BMD. Although not reaching statistical significance, there was an approaching main effect of HSAAR genotype on FBM% (*P* = 0.09) and LBM% (*P* = 0.08). HSAAR genotype had no significant interaction with endurance exercise or sex on body composition adaptation as visualized by DXA. However, although not reaching statistical significance, there was an approaching interactive effect of HSAAR genotype and endurance exercise on FBM% (*P* = 0.09) and BMC (*P* = 0.07).

### Endurance exercise decreased absolute and relative fat mass, but not total body mass or absolute lean mass

Rats in the 5 days/week training paradigm were comparatively leaner than sedentary rats after 9 weeks. TBM and FBM were significantly reduced in trained subjects compared to their sedentary counterparts (Fig. 2A and G; Table 3). Exercise across all subjects resulted in a leaner body composition wherein LBM% was significantly increased with a significant reduction in FBM% (Fig. 2B and D; Table 3). There was no observed main effect of endurance exercise on LBM, BMC, or BMD.

### Endurance exercise adaptations differed by sex in total body mass, lean mass, and bone mineral content, but not fat mass

A significant two-way interaction between sex and endurance exercise was observed (Fig. 2A–E and G; Table 4). Trained males had significantly lower TBM and BMC after 9 weeks of exercise, as well as higher LBM% compared to their sedentary male counterparts, however, LBM was not significantly different in trained males compared to their sedentary counterparts. In females, endurance exercise resulted in significantly higher LBM and LBM%, although there were no observed effects of endurance exercise on TBM or BMC. Exercising males had significantly lower FBM and FBM% compared to their sedentary counterparts. Likewise, exercising females had significantly lower FBM and FBM% compared to their sedentary counterparts. However, DXA measurements between sexes revealed that exercising males had significantly higher FBM, FBM%, LBM, TBM, and BMC than exercising females, yet no difference was found in LBM% across males and females after the 9-week endurance exercise paradigm. In comparison to trained females, sedentary males had higher FBM, FBM%, LBM, TBM, and BMC, yet had significantly decreased LBM%. Trained males had significantly higher LBM, TBM, and BMC compared to sedentary females. Subsequently, FBM% in trained males was significantly lower than that in sedentary females, however, there was no significant difference in FBM between endurance-trained males and sedentary females. Altogether, we note the sex-dependent and sex-independent body composition changes as a result of 9 weeks of endurance exercise in rats, of which none were influenced by muscle-specific AR overexpression. More specifically, endurance exercise elicits sex-independent losses in fat indices yet sex-dependent gains in female LBM, losses in male TBM, and losses in male BMC. These findings highlight the greater influence of whole-system sex signaling cascades, and the less likely influence of muscle-specific AR overexpression, on endurance exercise-mediated outcomes.

**Table 4:** Whole-body DXA measurements and changes by interactive effects of sex and endurance training

	*F* statistic (*df*)	Groups		*T* statistic	*P*-value
		Mean ± SEM	Mean ± SEM		
Fat body mass (g)	Sex × exercise *F*(1,81) = 20.52			–	< 0.001
		M-Ex: 14.63 ± 0.93	F-Ex: 6.51 ± 0.64	7.218	< 0.001
		F-Sed: 12.07 ± 1.29	F-Ex: 6.51 ± 0.65	3.868	< 0.01
		M-Sed: 38.19 ± 3.25	F-Ex: 6.51 ± 0.66	9.570	< 0.001
		F-Sed: 12.07 ± 1.29	M-Ex: 14.63 ± 0.93	− 1.611	n.s
		M-Sed: 38.19 ± 3.25	M-Ex: 14.63 ± 0.93	6.973	< 0.001
		M-Sed: 38.19 ± 3.25	F-Sed: 12.07 ± 1.29	7.473	< 0.001
Fat body mass %	Sex × exercise *F*(1,81) = 7.13			–	< 0.01
		M-Ex: 3.97 ± 0.22	F-Ex: 2.91 ± 0.26	3.093	< 0.05
		F-Sed: 5.59 ± 0.58	F-Ex: 2.91 ± 0.26	4.213	< 0.001
		M-Sed: 9.47 ± 0.73	F-Ex: 2.91 ± 0.26	8.417	< 0.001
		F-Sed: 5.59 ± 0.58	M-Ex: 3.97 ± 0.22	2.612	< 0.05
		M-Sed: 9.47 ± 0.73	M-Ex: 3.97 ± 0.22	7.170	< 0.001
		M-Sed: 9.47 ± 0.73	F-Sed: 5.59 ± 0.58	4.138	< 0.001
Lean body mass (g)	Sex × exercise *F*(1,81) = 5.71			–	< 0.05
		M-Ex: 341.37 ± 5.23	F-Ex: 208.02 ± 2.76	22.56	< 0.001
		F-Sed: 194.81 ± 2.96	F-Ex: 208.02 ± 2.76	− 3.262	< 0.01
		M-Sed: 349.38 ± 5.42	F-Ex: 208.02 ± 2.76	23.256	< 0.001
		F-Sed: 194.81 ± 2.96	M-Ex: 341.37 ± 5.23	− 24.390	< 0.001
		M-Sed: 349.38 ± 5.42	M-Ex: 341.37 ± 5.23	1.064	n.s
		M-Sed: 349.38 ± 5.42	F-Sed: 194.81 ± 2.96	25.037	< 0.001
Lean body mass %	Sex × exercise *F*(1,81) = 7.63			–	< 0.01
		M-Ex: 93.12 ± 0.23	F-Ex: 93.56 ± 0.25	− 1.304	n.s
		F-Sed: 90.91 ± 0.57	F-Ex: 93.56 ± 0.25	− 4.299	< 0.001
		M-Sed: 87.62 ± 0.73	F-Ex: 93.56 ± 0.25	− 7.738	< 0.001
		F-Sed: 90.91 ± 0.57	M-Ex: 93.12 ± 0.23	− 3.621	< 0.01
		M-Sed: 87.62 ± 0.73	M-Ex: 93.12 ± 0.23	− 7.211	< 0.001
		M-Sed: 87.62 ± 0.73	F-Sed: 90.91 ± 0.57	− 3.571	< 0.01
Total body mass (g)	Sex × exercise *F*(1,81) = 13.79			–	< 0.001
		M-Ex: 366.72 ± 5.89	F-Ex: 222.38 ± 3.11	21.679	< 0.001
		F-Sed: 214.37 ± 3.20	F-Ex: 222.38 ± 3.11	− 1.796	n.s
		M-Sed: 399.17 ± 6.59	F-Ex: 222.38 ± 3.11	24.248	< 0.001
		F-Sed: 214.37 ± 3.20	M-Ex: 366.72 ± 5.89	− 22.744	< 0.001
		M-Sed: 399.17 ± 6.59	M-Ex: 366.72 ± 5.89	3.670	< 0.01
		M-Sed: 399.17 ± 6.59	F-Sed: 214.37 ± 3.20	25.219	< 0.001
Bone mineral content (g)	Sex × exercise *F*(1,81) = 15.75			–	< 0.001
		M-Ex: 10.73 ± 0.19	F-Ex: 7.87 ± 0.13	12.366	< 0.001
		F-Sed: 7.48 ± 0.09	F-Ex: 7.87 ± 0.13	− 2.470	n.s
		M-Sed: 11.60 ± 0.18	F-Ex: 7.87 ± 0.13	16.593	< 0.001
		F-Sed: 7.48 ± 0.09	M-Ex: 10.73 ± 0.19	− 15.470	< 0.001
		M-Sed: 11.60 ± 0.18	M-Ex: 10.73 ± 0.19	3.277	< 0.01
		M-Sed: 11.60 ± 0.18	F-Sed: 7.48 ± 0.09	20.316	< 0.001

F-Ex, exercising female (*n* = 14); M-Ex, exercising male (*n* = 23); F-Sed, sedentary female (*n* = 24); M-Sed, sedentary male (*n* = 23). Multiple comparisons data presented here were analyzed by post hoc Bonferroni-corrected *t*-tests when indicated by three-way LMER ANOVA. Non-significant sex by exercise interactive main effects not shown, see Additional file 1: Tables S2 and S3

### MHC2a and MHC1 expression differed by sex only in tibialis anterior, whereas MHC2a expression differed by HSAAR genotype only in soleus

**Fig. 3:**
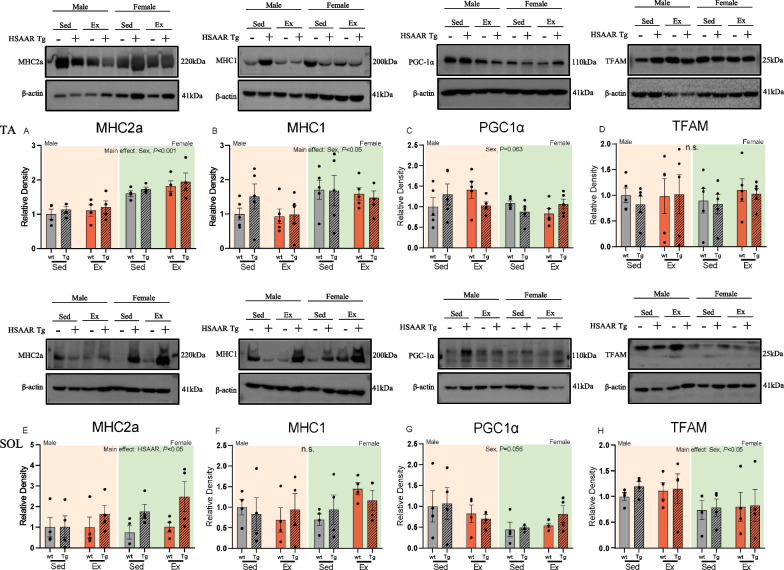
The effect of sex and HSAAR Tg genotype on glycolytic and oxidative muscle fiber-type expression and canonical mitochondrial biogenesis protein marker expression. Quantification of western blot analysis of **a**, **e** myosin heavy chain 2a (MHC2a), **b**, **f** myosin heavy chain 1 (MHC1), **c**, **g** peroxisome proliferator-activated receptor gamma coactivator 1 alpha (PGC1α), and **d**, **h** mitochondrial transcription factor A (TFAM) from tibialis anterior (**a**–**d**) and soleus (**e**–**h**) muscles. Representative immunoblots for tibialis anterior (**a**–**d**) and soleus (**e**–**h**) are shown between males and females, with their respective exercise and genotype groups. Indicated groups: males (*yellow*), females (*green*), sedentary (*grey*), aerobic exercise (*red*), wild-type (wt) (*blank fill*), and HSAAR transgenic (Tg) (*hatch fill*). Data expressed in relative density (R.D.) and presented as means ± s.e.m. Data analyzed using three-way ANOVA for (**a**–**h**). **b**–**d** *n* = 5 per sex–genotype–exercise group, **a**, **e**–**h** *n* = 4 per sex–genotype–exercise group

Following the 9-week paradigm, TA and SOL were dissected and prepared for immunoblotting as they are predominantly glycolytic and oxidative muscles in rodents, respectively. Sex presented as a main effect factor in glycolytic TA, wherein females had significantly higher MHC2a protein expression than their male counterparts (F: 1.77 ± 0.08, M: 1.11 ± 0.07, *F*_(1,37)_ = 36.45, *P* < 0.001) (Fig. 3A). Neither the HSAAR genotype nor its interaction between sex and/or endurance exercise influenced MHC2a expression in glycolytic TA. In oxidative SOL, there were no observed effects of either sex or endurance exercise on MHC2a expression. However, a main effect of the HSAAR genotype on MHC2a expression was found in SOL, wherein transgenic rats expressed higher levels of MHC2a regardless of their sex or exercise status (Tg: 1.72 ± 0.27, WT: 0.93 ± 0.18, *F*_(1,29)_ = 5.64, *P* < 0.05) (Fig. 3E). In TA, MHC1 expression differed by sex, where females had higher expression than males (F: 1.61 ± 0.14, M: 1.11 ± 0.13, *F*_(1,37)_ = 6.43, *P* < 0.05) (Fig. 3B). However, TA MHC1 expression was not affected by the HSAAR gene or exercise alone, or by the interactive effects of sex, HSAAR expression, and endurance exercise. Likewise, neither sex, HSAAR expression, nor training was observed to affect MHC1 expression in SOL (Fig. 3F). Altogether, we present that female rats express greater Type 1 and Type 2a fiber type in predominantly glycolytic limb muscle and highlight the HSAAR-dependent effect of Type 2a in predominantly oxidative limb muscle. We observe that muscle-specific AR overexpression may promote fast-twitch fibers in otherwise oxidative soleus in a sex- and endurance-exercise-independent manner.

### TFAM expression in soleus was elevated in males compared to females

Considering the significantly higher mitochondrial enzyme activity in skeletal muscle dissections of HSAAR males in Fernando et al. [52], we examined the effects of sex, HSAAR genotype, and endurance exercise on the master regulator of mitochondrial biogenesis, PGC1α, and its downstream targets, TFAM and NRF-2 [62–64]. In glycolytic TA, ANOVA revealed an approaching significant effect of sex on PGC1α expression (*P* = 0.063). There was a significant three-way interaction in tibialis PGC1α expression across sex, HSAAR genotype, and exercise groups (*F*_(1,37)_ = 6.09, *P* < 0.05) (Fig. 3C). Although no individual groups differed in a recognizable pattern in the post hoc analysis, the ANOVA interaction appeared such that HSAAR reverses the sex differences in exercise-induced PGC1α response. In Fig. 3C, PGC1α protein appeared elevated in trained wild-type males but reduced in trained wild-type females, and these sex differences seemed to reverse with the presence of HSAAR. Specifically, PGC1α protein trended lower in trained HSAAR males but appeared elevated in trained HSAAR females. In TA, expression of TFAM (Fig. 3D) was not affected by sex, HSAAR genotype or chronic endurance exercise independently or interactively. In SOL, there was a main effect of sex on TFAM expression wherein males had higher whole-muscle expression than females (F: 0.79 ± 0.11, M: 1.12 ± 0.08, *F*_(1,29)_ = 4.73, *P* < 0.05) (Fig. 3H). Although not reaching significance, the three-way ANOVA revealed a trend towards a main effect of sex on PGC1α expression in SOL (*P* = 0.056) (Fig. 3G). There was no observed main or interactive effect of endurance exercise or HSAAR genotype on PGC1α, TFAM, or NRF-2 expression in SOL. Neither TA nor soleus exhibited changes to NRF-2 expression by sex, HSAAR genotype, or chronic training (Additional file 1: Figs. S1 and S2). Altogether, we show no effect of muscle-specific AR overexpression on the protein expression of mitochondrial biogenesis regulators in tibialis anterior or soleus despite the striking increase in mitochondrial ETC activity seen in male HSAAR rat EDL in previous work by Fernando et al. [52].

### HSAAR expression promotes glycolytic myofiber size, but its presence diminishes size sex differences in oxidative myofiber

**Fig. 4:**
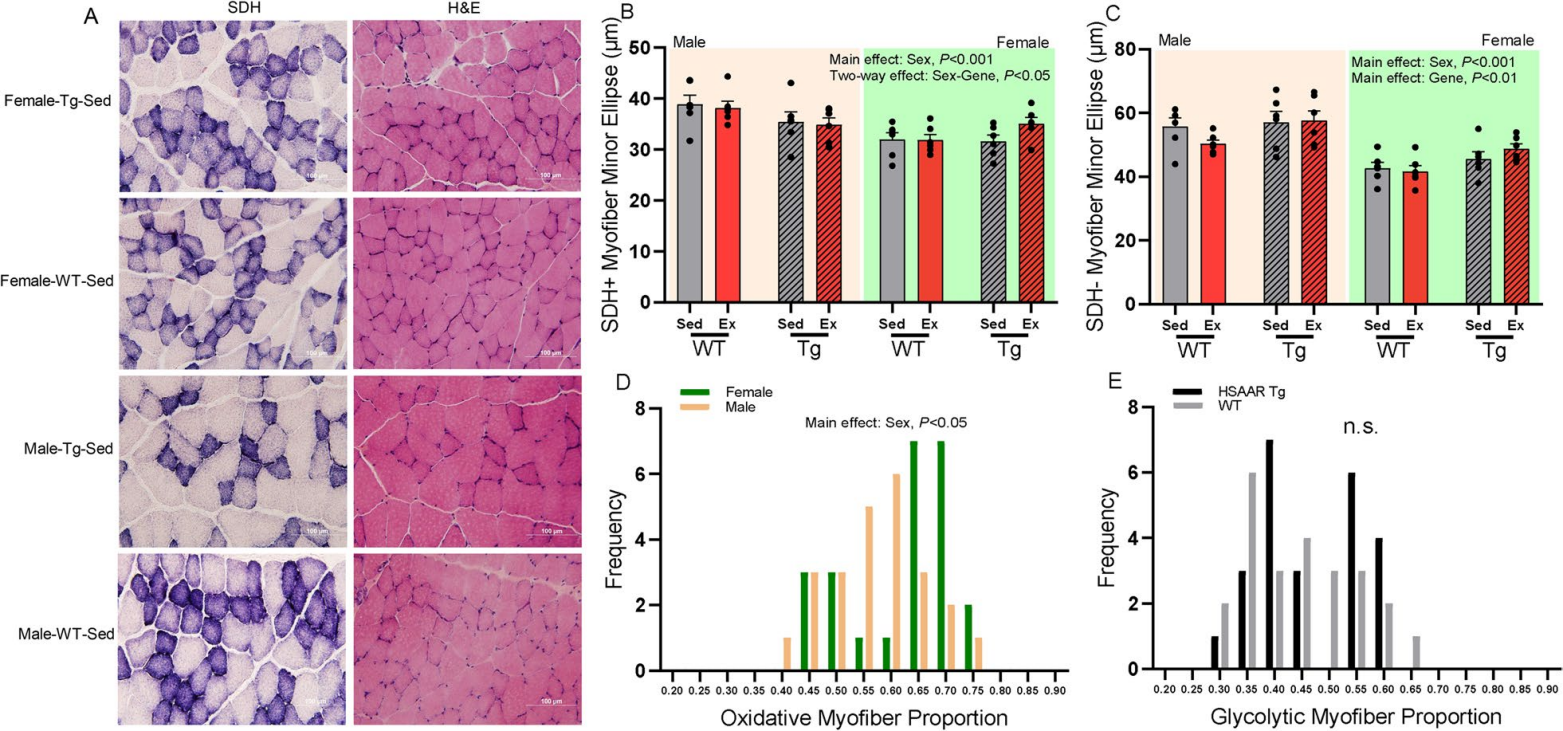
HSAAR Tg genotype affects glycolytic myofiber size but not proportion, while sex affects both glycolytic and oxidative myofiber size and proportion across EDL. Visualization of **a** succinate dehydrogenase (SDH) stained, hematoxylin and eosin (H&E) matched transverse photomicrographs of extensor digitorum longus (EDL) at 40× magnification, **b** quantification of minor ellipse of oxidative SDH-stained H&E-matched myofibrils, **c** quantification of minor ellipse of glycolytic SDH-nonstained H&E-matched myofibrils, **d** proportion of oxidative (SDH-stained) myofibrils between male and female rats across 40× magnified photomicrographs (collapsed by genotype and exercise), **e** proportion of glycolytic (SDH-nonstained) myofibrils between HSAAR Tg and WT rats across 40× magnified photomicrographs (collapsed by sex and exercise). Photomicrographs (**a**) represent between-group subjects across SDH and H&E staining after the experimental paradigm. Indicated groups (**b**, **c**): males (*yellow*), females (*green*), sedentary (*grey*), aerobic exercise (*red*), wild-type (wt) (*blank fill*), and HSAAR transgenic (Tg) (*hatch fill*). Data (**b**, **c**) are presented as means ± s.e.m, with *n* = 6 per sex–genotype–exercise group. Data (**d**, **e**) are presented as frequency distributions. **d** *n* = 24 per sex (collapses *n* = 6 transgene–exercise, *n* = 6 wild-type–exercise, *n* = 6 transgene–sedentary, *n* = 6 wild-type–sedentary). **e** *n* = 24 per genotype (collapses *n* = 6 female–exercise, *n* = 6 male–exercise, *n* = 6 female–sedentary, *n* = 6 male–sedentary). All data analyzed using three-way ANOVA with post hoc Tukey HSD for multiple comparisons. Scale bar, 100 µm

Succinate dehydrogenase (SDH) enzymatic staining of EDL transverse sections with nitro-blue tetrazolium was used to qualify oxidative and glycolytic myofibers (SDH+ and SDH−, respectively). The myofibers were overlayed to their H&E-matched sections to quantify oxidative and glycolytic myofiber size. Minor ellipse was quantified to approximate myofiber size rather than CSA due to minor ellipse estimates better resisting bias due to variability in fiber orientation (i.e., it does not assume a perpendicular cross-section). Minor ellipses of both oxidative and glycolytic myofibers were larger in males compared to females (SDH+, F: 32.61 ± 0.65 µm, M: 36.82 ± 0.84 µm, *F*_(1,45)_ = 16.85, *P* < 0.001; SDH−, F: 44.68 ± 1.05 µm, M: 55.17 ± 1.41 µm, *F*_(1,45)_ = 40.03, *P* < 0.001.) (Fig. 4B and C, respectively). Although males showed greater oxidative and glycolytic myofiber size in EDL, the proportion of SDH+ stained myofibers in EDL was greater in females, than males, presenting a clear sex difference in myofiber oxidative state (SDH+, F: 0.59 ± 0.02, M: 0.54 ± 0.02, *F*_(1,45)_ = 4.21, *P* < 0.05; Fig. 4D). Subsequently, males showed greater proportion of glycolytic (SDH−) myofibers (Additional file 1: Fig. S3A). There was an observed two-way effect of sex and HSAAR genotype on minor ellipse length of oxidative myofibers, wherein wild-type males had greater minor ellipse length than either HSAAR or wild-type females (M-WT: 38.49 ± 1.07 µm, F-Tg: 33.34 ± 0.99 µm, *P* < 0.01; M-WT: 38.49 ± 1.07 µm, F-WT: 31.89 ± 0.83 µm, *P* < 0.01; Fig. 4B). However, once males expressed HSAAR, there were no differences in oxidative minor ellipse lengths between HSAAR males and females, suggesting that sex differences in oxidative myofiber size are dampened when males express the HSAAR transgene. Interestingly, the proportion of glycolytic (SDH−) myofibers did not change in response to the HSAAR transgene (Fig. 4E), even though HSAAR expression did significantly increase glycolytic myofiber minor ellipse size (Tg: 52.25 ± 1.65 µm, WT: 47.60 ± 1.52 µm, *F*_(1,45)_ = 7.84, *P* < 0.01; Fig. 4C). There were no effects of endurance training in SDH-staining and oxidative proportion in EDL, nor on minor ellipse length (Additional file 1: Fig. S3C and D). Altogether, these results point to a mechanism wherein transgenic myocytic AR overexpression may favor glycolytic transition by selectively increasing glycolytic myofiber size, but not their overall count.

### In trained rats, glycolytic muscle size, but not whole-body composition, is correlated with levels of whole-muscle AR content

**Fig. 5:**
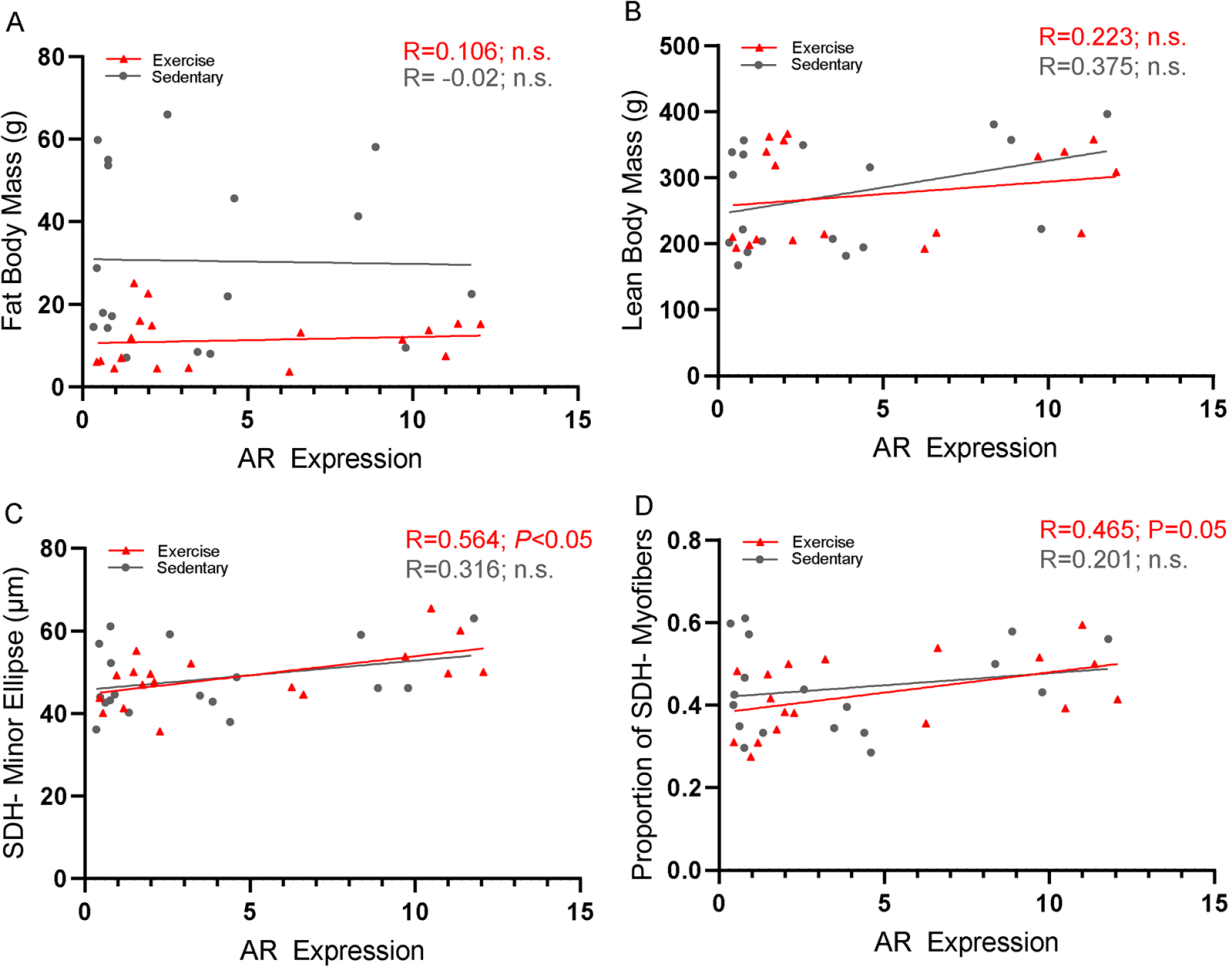
Trained rats show moderate correlation between glycolytic muscle androgen receptor expression and glycolytic myofiber size, but not whole-body composition. Correlations are presented in panels for: **a** DXA-measured fat body mass, **b** DXA-measured lean body mass, **c** succinate dehydrogenase nonstained (SDH-) glycolytic myofiber minor ellipse in extensor digitorum longus (EDL), and **d** proportion of SDH- glycolytic myofibers in EDL. Data are presented as subject-matched AR expression (relative density, R.D.) and measured outcome variables, and all correlations are analyzed using Pearson’s correlation. Data are stratified by exercise (*red, closed triangles*) and sedentary (*grey, closed circles*) groups, with sex and genotype groups collapsed (**a**–**e**). *n* = 18 per exercise group (collapses *n* = 4 female–transgene, *n* = 5 female–wild-type, *n* = 4 male–transgene, *n* = 5 male–wild-type), and *n* = 18 per sedentary group (collapses *n* = 4 female–transgene, *n* = 5 female–wild-type, *n* = 4 male–transgene, *n* = 5 male–wild-type)

The significant independent effects of HSAAR and endurance exercise on body composition measurements of lean and fat mass, together with the significant main effects of HSAAR expression on glycolytic size, suggested that AR expression in muscle might mediate body composition and muscle phenotype changes after exercise. In Fig. 5, we show Pearson correlations collapsed by exercise and sedentary groups across tibialis anterior AR relative densities and fat body mass (Fig. 5A), lean body mass (Fig. 5B), glycolytic minor ellipse (Fig. 5C), and glycolytic myofiber proportion (Fig. 5D). Consistent with this hypothesis, significant Pearson correlations were observed between whole-muscle AR expression and glycolytic minor ellipse across endurance-trained rats (*R* = 0.593, *P* < 0.01, Fig. 5C). Further, although not reaching statistical significance, there was a trend towards a moderate correlation between whole-muscle AR expression and glycolytic myofiber proportion across endurance-trained rats (*R* = 0.465, *P* = 0.05, Fig. 5D). Altogether, greater expression of AR in limb skeletal muscle of trained, but not sedentary rats, contributes more to improved myofiber phenotype, and less so to whole-body lean and fat body parameters.

## Discussion

Here, we present a sex-balanced investigation on the effects of muscle-specific AR overexpression and chronic, forced endurance exercise in rodent body composition and skeletal muscle. Using this model, we show that the muscle-specific AR overexpression transgene is sex-dependent, as AR expression is approximately fivefold and tenfold greater in transgenic females and males, respectively, compared to wild-types. Consistent with our previous work, we found that muscle-specific overexpression of AR promotes lean body composition and greater glycolytic myofiber size without sex-specific effects. We extend these findings to showcase the distinct sex differences in adaptation to chronic endurance exercise in lean, total, and bone mass, as well as the sexual difference present in myofiber size and proportion of classified oxidative and glycolytic muscle. After a total of 9 weeks of chronic endurance training, we found that muscle AR was elevated after 24 h following the last bout of exercise and that only endurance-trained rats presented a moderate correlation between increased myocytic AR expression and glycolytic myofiber size.

Masculinizing sex characteristics in body composition are intrinsically linked to physiological testosterone secretion and normal AR functioning [1, 65, 66]. Decades of research highlight global recreational use of synthetic anabolic testosterone by both male and female adolescents and adults with overall goals of improving body composition [67–73]. However, several studies question the causal relationship of increased testosterone and muscle hypertrophy or performance and instead point to the expression of AR as promoting exercise-mediated muscle size [54, 58] and exercise performance [36, 37, 39, 41, 43, 45, 46, 50, 74]. In mice, sex-dependent effects of forced and voluntary running on body composition are noted [75–77], yet the reliance of male subjects under sedentary lifestyle in transgenic AR literature limits knowledge of muscle-specific AR actions across both sex and exercise. To explore this question, we examined sex effects on body composition and muscle phenotype in response to chronic, forced endurance exercise in transgenic muscle-specific AR overexpression rats.

Although it is well established that females have lesser skeletal muscle mass and body weight when age-matched to males in both rodent [78, 79] and human [80, 81] studies, these differences are affected by endurance exercise [76, 82]. Here we find an interaction between sex and endurance exercise where males always maintained greater lean mass, fat mass, body mass, and bone mineral content than their trained or sedentary female counterparts. We observed sex differences such that trained males, compared to sedentary males, had reduced bone mineral content, reduced body weight, yet had no change in lean mass. However, compared to sedentary females, the trained females exhibited no change in bone mineral content or bodyweight yet showed increased lean body mass. Endurance-training reductions in body weights of male but not female rats have been shown previously [75–77]. Interestingly, trained males were able to reduce their fat mass to levels no different than sedentary females, all while lowering their body fat percentage beyond that of sedentary females. This can likely be interpreted by the higher overall body weight and adipose tissue available for exercise-mediated lipolysis in males, which together contribute to their decreased fat percentage. This phenomenon has been reported in a study of a 20-week endurance exercise paradigm between healthy men and women, where adipocyte epinephrine-stimulated lipolysis responds at better rates in trained males compared to trained females, with significantly more fat reduction in males compared to females [83].

Contrary to expectation, in this investigation we did not observe a statistically significant effect of muscle-specific AR overexpression on reducing indices of fat mass in either sex. Previously, an HSAAR transgene effect on fat mass was observed in Fernando et al. [1] wherein 6-, 8-, and 10-week-old male HSAAR rats had significantly lower absolute and relative fat body mass compared to their age-matched wild-type counterparts. This disparity is likely due to differences in study design, mainly the age during captured DXA scans. Here, rats began the exercise or sedentary paradigms at PND60–90 (i.e., 8–12 weeks of age) and were terminally DXA scanned 9 weeks later (i.e., 4–5 months old). Unlike the juvenile, peripubertal, and adolescent developmental periods captured by repeated DXA scanning in Fernando et al., we highlighted the body composition of the young adult rat [40]. Rapid gain in body mass of Sprague Dawley rats is seen until PND168 (i.e., 5.5 months old) only reaching a plateau beyond 12 months of age [41], suggesting that manipulation of muscle-specific AR may likely have stronger effects on fat mass accretion during the earlier phases of the rapid growth period of adolescence and adulthood. Next works should investigate the lifespan impact of muscle-specific AR overexpression on male and female body composition at distinct developmental time points to investigate this hypothesis.

Sex differences in serum concentration of total and free testosterone are unequivocal, yet intramuscular AR expression between male and female rodents or humans are more ambiguous. In wild-type and sedentary mice, AR gene expression in gastrocnemius was no different between males or females at 4- and 12 weeks old [44]. In resistance-trained humans, females can have similar [26] or significantly lower levels [25] of AR protein in vastus lateralis, compared to their male counterparts. Androgen receptor content across the HSAAR transgenic rodent was significantly higher in both male and female groups compared to their wild-type littermates (Fig. 1A) and although there were no sex differences in intramuscular AR between wild-type rats, the presence of the HSAAR transgene drove male intramuscular AR higher. Regardless of this sexually dimorphic effect on intramuscular AR, the HSAAR genotype was still found to increase lean body mass (Fig. 2C) and glycolytic myofiber size (Fig. 4C) across both sexes, although sex differences were conserved across male and female lean body mass and glycolytic myofiber size. Interestingly, this contrasts the work of Fernando et al. [52] using rats and Musa et al. [84] using mice where only testosterone-treated HSAAR females replicated the skeletal muscle phenotype of untreated HSAAR males, pointing towards the reliance of disparate circulating androgen levels in the promotion and maintenance of skeletal muscle sex differences. Androgen receptor reliance on circulating androgens both for genomic and non-genomic effects is indisputable, and so seminal vesicle weights of HSAAR rats were assessed as an estimate of serum testosterone levels [64]. As seen in Fig. 2H, seminal vesicle mass was neither changed by HSAAR Tg expression in male rats, nor by 9 weeks of endurance exercise. In previous AR overexpression literature, serum testosterone concentration was also unaltered in male mice with osteoblast AR overexpression [14]. Concerning exercise-mediated effects of androgens, other studies provide evidence to suggest that circulating androgens are less predictive of resistance training-induced changes to body composition and muscle phenotype [85, 86]. Although immediate increase in serum testosterone after an acute bout of exercise is often observed in both men and women [67–70], it is not a consistent finding [10, 71, 72] and the hypothesis that it contributes to accretion of muscle mass, fiber-type switching, CSA increase, or myofibrillar protein synthesis is highly controversial [66, 73]. This debate stems primarily from the difficulty of assessing the causal impact of transiently elevated plasma testosterone on the long term, eventual changes seen in myofiber phenotype after chronic exercise. However, both long-term and acute exercise-mediated fluctuations in free testosterone, DHT, and steroid hormone binding globulin (SHBG) are highly variable to the implemented exercise paradigm (aerobic or resistance), the intensity of exercise, and the participant demographic (age, sex, or trained/untrained) [74, 75]. On the basis of sex, puberty-mediated elevations in testosterone in males (from < 7–8 nmol/L to 15–20 nmol/L) drastically diverge from the circulating levels seen in females (< 2 nmol/L) [76]. Prior to this, there are minimal sex differences in performance across running and jumping sports, however post-pubertal males outperform their age-matched female counterparts, likely due to the dramatic difference in circulating androgens and their indirect and direct impact on other tissues, such as muscle and bone [5].

We show that muscle-specific AR overexpression increases lean mass in both sexes (Fig. 2C) and provides evidence to support hypotheses of female responsiveness to intramuscular AR activity with physiologically relevant circulating androgen levels. However, endurance training only increases lean mass in females, and we found no interaction of exercise and HSAAR expression, suggesting that despite a similar lean phenotype outcome, exercise and HSAAR expression may regulate body composition using distinct mechanisms. If the HSAAR transgene consistently elevates muscle-specific AR protein levels throughout the lifespan of HSAAR rats compared to wild-type rats, we can speculate that the possible lifelong 5- and 10-fold increase in skeletal muscle AR in females and males, respectively, is likely a more potent stimulator of lean mass accretion than endurance training alone, where intensity, frequency, modality, and resultant downstream signaling cascades may likely be more influenced by sex expression [75–77, 87–90]. The shift in metabolic demand from endurance training over a period of several weeks may influence canonical exercise signaling pathways [91], likely in a sex-specific manner, but remains an acute treatment compared to the chronic influence of the HSAAR transgene. In sedentary transgenic knockout models, muscle phenotype and lean mass is not effected by ARKO or sex in muscle-ARKO mice [92], suggesting that muscle-specific AR may not be absolutely required for normal development of skeletal muscle in males or females. However, ablation of myoblast, myocyte, or myofiber AR can disrupt skeletal muscle mass and key regulators of protein synthesis in male mice [48, 93], supporting the hypothesis that the lifelong influence of lost or overexpressed skeletal muscle AR effects molecular pathways regulating muscle phenotype and metabolism in a sex-independent manner.

Although exercise adaptations and performance have yet to be causally related to muscle-specific AR conclusively, Morton et al. [58] proposed that pre-exercise levels of intramuscular AR in males, but not steroid hormones, were strongly correlated to larger resistance-training adaptations to lean mass as well as Type 2a and Type 1 CSA. We were interested in identifying a relationship between muscle-specific AR expression and endurance exercise-related adaptations. When subjects were collapsed by sex, there was a moderate correlation between levels of intramuscular AR and glycolytic myofiber size and proportion in only exercising rats (Fig. 5C, D), with no such relationship seen between intramuscular AR and whole-body composition outcomes within exercise or sedentary rats. Recent work from Yin et al. [54] found that transient inhibition of global AR function by flutamide treatment reversed both endurance and resistance training-induced gains in muscle mass in male rats. Pharmacological inhibition of AR downregulated exercise-induced protein expression of intramuscular AR, mTOR, IGF-1, IGF-1R, p-PI3K, and p-Akt in oxidative soleus during endurance training and, respectively, in glycolytic gastrocnemius during resistance training. Our results echo that exercise increases intramuscular AR protein, where Yin et al. extend their hypothesis that this exercise-mediated AR increase activates hypertrophic mTOR signaling pathways to promote lean mass hypertrophy. Although natal males have been the predominant focus of media and policy concerning androgen interventions in exercise adaptation therapeutics, these interventions are also relevant to natal females, who can experience conditions of androgen excess either endogenously (e.g., polycystic ovarian syndrome), or exogenously due to abuse of exogenous androgen in sport androgen treatment clinically (e.g., masculinizing hormone therapy). Although androgen-based treatment of muscle wasting conditions has been avoided in natal females due to concerns of masculinizing side effects, our finding of sexually equivalent muscle AR expression across wild-type rats, and sex-independent accretion of lean mass in response to muscle-specific AR overexpression suggest that selective androgen receptor modulators (SARMs) targeting skeletal muscle may be efficacious for this purpose.

Muscle AR seems to contribute to the maintenance of fast-twitch fibers in male mice [48, 94, 95], however neither endurance-trained HSAAR rats in this work nor sedentary HSAAR rats in Fernando et al. 2010 demonstrated an oxidative or glycolytic fiber-type switch in dissected EDL. We extended our analyses by including dissected oxidative soleus and found that HSAAR increased Type 2a expression in soleus (Fig. 3F) and mediated glycolytic myofiber hypertrophy in EDL (Fig. 4C). This androgenic-mediated fast-to-slow fiber type effect is presented in work from Isayama et al. [96] where testosterone treatment increased CSA of Type 1 fibers in soleus and Type 2a fibers in EDL. Additionally, we highlight a major sex effect with greater oxidative expression of Type 1 and Type 2a protein found in female glycolytic TA muscle (Fig. 3A and B), corresponding with the increased proportion of oxidative myofibers in EDL (Fig. 4D). This sex difference in oxidative myofiber size and proportion is well-established [4, 97] and although this paradigm and data collection did not address endurance capacity between groups, we can interpret that the larger oxidative myofiber presence measured by immunoblotting and histological staining in females is supportive of the hypothesis of greater exercise capacity in females, both in rodent and human work [78, 98]. It is well documented that fiber type transition can occur between Type 1 and Type 2a in skeletal muscle as a response to the metabolic demands of exercise [99, 100]. Although our results did not present endurance exercise-induced fiber type adaptations, it is important to recognize the limitations of qualifying an endurance exercise response due to lack of performance standardization by individual maximal oxygen uptake (V̇O_2_max) testing or power output measures. The training paradigm used here was adapted by the protocol published by Smolka et al. [60] using rats, and later adapted by Fontana et al. [56] using mice, where they showed their endurance training protocol interacted with mesterolone treatment to increase TA Type 1 and Type 2a fiber percentages. We can speculate that differences in results between our works could be due to species and modality differences, as our work utilized a forced running wheel apparatus which may result in different metabolic adaptations in skeletal muscle than that seen in treadmill running [101].

To explain the significant increase in Complex I–IV activity in HSAAR transgenic males in our previous work [52], we examined several markers of mitochondrial biogenesis. Metabolic regulator PGC1α and its transcription factor target NRF-2 are known to stimulate the expression of TFAM and nuclear-encoded mitochondrial genes upon activation by AMPK during states of high energetic demand [62, 63]. We predicted that the HSAAR transgene and chronic endurance training would synergistically increase protein expression of the PGC1α pathway, however, we show no change of whole-muscle protein expression of PGC1α, NRF-2 (Additional file 1: Tables S4 and S6) or TFAM by neither transgenic AR overexpression nor the 9-week endurance paradigm. We present a sexual difference in higher expression of TFAM in male soleus, which contrasts other work highlighting greater female mtDNA, TFAM content, and OXPHOS activity [102]. Some human literature seems to point to greater abundance, size, and functioning of mitochondria in females [103] yet others point to no sex effects in beta-oxidation enzyme activity, mitochondrial size, or abundance [104]. Our use of whole muscle and not nuclear compartments may have limited our observation of catabolic exercise stimuli or AR overexpression-facilitated activation of the PGC1α pathway. Additionally, as we only assessed the main mitochondrial biogenesis regulators, it is possible that other gene targets of mitochondrial function may be at play in the HSAAR rat [52].

Although mechanical stimulus has beneficial effects on the plasticity of bone properties and microstructure, these effects vary by exercise type, intensity, and duration because these factors determine the nature of the applied mechanical stimuli. We observed a reduction in male BMC as a result of the 9 weeks of forced wheel running (Fig. 2E), which is not entirely unexpected in light of reported decreases in bone quality with excessive mechanical stimulus. For example, male rats undergoing forced treadmill running at 20 m/min have no improvement in trabecular bone volume or BMD, while these measures are significantly higher in those running at a lower intensity of 12 m/min, highlighting the impact of varying exercise intensity on bone [61]. Similarly, male rats training at 22–30 m/min for 9 weeks exhibit a reduction in proximal tibia BMD, decreased trabecular thickness, and increased trabecular separation [62]. Additionally, there are observed increases in proinflammatory cytokines after 8 weeks of downward slope vs. upward slope treadmill running to exhaustion in male mice, which further highlights the impact of intensity and treadmill type on adverse effects of exercise [63]. Altogether, with the decrease in male BMC and the unaltered BMC in female rats (Table 4), it is possible that the implemented protocol and wheel design, although effective in producing expected exercise adaptations in lean and fat mass, may have provided mechanical stimulus in excess of that which encourages bone anabolism.

Although forced exercise paradigms are highly advantageous in their ability to match rodent exercise patterns to that seen in humans, there are concerns about their impact on glucocorticoid-mediated stress responses and muscle catabolism. As steroid hormones, glucocorticoids can directly induce the transcription of muscle-wasting genes under excessive or prolonged activation of glucocorticoid receptors [81]. Prolonged time of single-bout forced treadmill sprints can elevate serum corticosterone levels in untrained male and female mice, with females exhibiting a more rapid elevation at the onset of exercise than males [82]. In endurance-trained humans, males show a plasma cortisol increase only when training at 80% of maximum heart rate for 120 min, but not when training at 50% maximum heart rate for 20 min [83]. In contrast, recent work from Salamone et al. [84] shows that weekly glucocorticoid treatment in mice increases ATP production, maximal tetanic and specific force production, and contraction speed in both sexes. Additionally, the weekly glucocorticoid exposure alters the skeletal muscle transcriptome in a sex-specific manner where genes regulating muscle hypertrophy are elevated in males and genes upregulating lipid utilization are elevated in females. Although the mechanisms by which glucocorticoids regulate muscle anabolism across sex or exercise modalities are complex, we suspect that acute fluctuations in glucocorticoids from the many psychological and physiological stresses of forced treadmill exercise influence muscle, fat, and bone plasticity to exercise. Further investigation is needed to identify if glucocorticoid receptors are directly impacted by the HSAAR transgene, and their roles in sex-specific exercise adaptations.

It is well established that AR and testosterone regulate primary and secondary sex characteristics and body composition as well as contributing phenotypic and metabolic responses alone or in combination with resistance or endurance exercise. While we were able to highlight these sex differences in body composition with or without exercise treatment, our work failed to identify any interactions between exercise and HSAAR genotype, or sex, exercise, and HSAAR genotype. First, we speculate that some of this may be due to: physiologically lower concentrations of circulating testosterone in females [52]; the use of an exercise paradigm developed from data in male subjects, which may be insufficient for female androgenic adaptations [56, 60]; or acute dysregulation in hypothalamic secretions to maintain reproductive fitness in response to overly strenuous exercise [105, 106]. Second, we recognize the temporal regulatory ability of AR ubiquitously across tissues and its dynamic range of expression when exposed to environmental stimuli, such as exercise. Additionally, the promoter regulation of the endogenous AR gene is likely different than that which is present in the HSAAR rat. For example, even when we genetically force overexpression of intramuscular AR, there are still individual differences in AR expression (Fig. 5) which raise the possibility of homeostatic ceilings for AR-mediated adaptation in HSAAR males and females. Individual variation in male human skeletal muscle AR expression before resistance training was shown to dictate the level of Type 1 and 2 fiber size and overall change in lean mass after 12 weeks of resistance exercise [58]. This work tested male subjects within physiological ranges, while our work speaks to a slightly different hypothesis where we focus on supraphysiological AR content in rats using a high-intensity endurance paradigm. Due to the plastic nature of various tonic stimulation on the metabolic and contractile properties in skeletal muscle, there are many possibilities for future exercise work to focus on the specific biology that regulates AR within HSAAR mutants. The HSAAR transcriptional regulation of AR and hypertrophy-related genes activated through androgen-AR binding can be understood further using Tfm mutants and HSAAR/Tfm crosses to parse out effects of global AR dysfunction and possible muscle-specific AR rescue in rats, respectively.

## Perspectives and significance

In summary, we conclude that, despite surface similarity in phenotype, long-term endurance training adaptations do not appear to interact with myocytic AR overexpression, suggesting that intramuscular AR and exercise affect body composition and muscle hypertrophy via distinct mechanisms of action. We observed both sex-dependent and sex-independent effects of both exercise and AR overexpression, which also did not appear to be affected by AR overexpression. The overexpression paradigm allows us to evaluate sufficiency, but it does not allow us to rule out that there is a necessary function of intramuscular AR in sexually differentiated exercise adaptation. It is intriguing that exercise increased intramuscular AR and did so similarly in both sexes. This change is consistent with androgenic milieu being important for exercise response, but that there is an asymptote on the effect of increasing AR expression in muscle, such that overexpression does not further affect response. These findings extend our current knowledge regarding the whole-body and molecular effects of overexpressed AR in skeletal muscle under chronic, forced endurance exercise in a sex-balanced cohort, and highlight the need for further exploration into the dynamic regulation of AR within muscle during exercise, and mechanisms involved in AR- and exercise-mediated anabolism.

## Supplementary Information

**Additional file 1: Table S1. **Supplemental statistics for exclusion criteria of exercising females. **Table S2.** Supplemental LMER ANOVA statistics for Fig. 2. **Table S3.** Supplemental post hoc Bonferroni-corrected *t*-test statistics for Fig. 2. **Table S4.** Supplemental ANOVA statistics for Figs. 1A and 3A–D in TA. **Table S5.** Supplemental post hoc Tukey HSD statistics for Fig. 1A and 3C in TA. **Table S6.** Supplemental ANOVA statistics for Fig. 3E–H in SOL. **Table S7.** Supplemental ANOVA statistics for Fig. 4B, C in EDL. **Table S8.** Supplemental post hoc Tukey HSD statistics for Fig. 4B in EDL. **Table S9.** Supplemental ANOVA Statistics for Fig. 4D, E in EDL. **Table S10.** Supplemental Pearson correlation statistics for Fig. 5. **Figure S1.** Quantification of western blot analysis of nuclear respiratory factor 2 (NRF-2) in TA. **Figure S2.** Quantification of western blot analysis of nuclear respiratory factor 2 (NRF-2) in SOL. **Figure S3.** Glycolytic (SDH−) and oxidative (SDH+) myofiber count proportions across EDL and the effects of sex, HSAAR genotype, or endurance exercise on their distribution.

## Data Availability

The data sets used during the current study are available from the corresponding author upon reasonable request.
