# Supplementary material for: Myocytic androgen receptor overexpression does not affect sex differences in adaptation to chronic endurance exercise
Source: Biol Sex Differ. 2022 Oct 23;13:59. doi: 10.1186/s13293-022-00471-x (PMC9590152; doi:10.1186/s13293-022-00471-x)
Supplement: Supplementary file 1 — Additional file 1: Table S1. Supplemental statistics for exclusion criteria of exercising females. Table S2. Supplemental LMER ANOVA statistics for Fig. 2. Table S3. Supplemental post hoc Bonferroni-corrected t-test statistics for Fig. 2. Table S4. Supplemental ANOVA statistics for Figs. 1A and 3A–D in TA. Table S5. Supplemental post hoc Tukey HSD statistics for Fig. 1A and 3C in TA. Table S6. Supplemental ANOVA statistics for Fig. 3E–H in SOL. Table S7. Supplemental ANOVA statistics for Fig. 4B, C in EDL. Table S8. Supplemental post hoc Tukey HSD statistics for Fig. 4B in EDL. Table S9. Supplemental ANOVA Statistics for Fig. 4D, E in EDL. Table S10. Supplemental Pearson correlation statistics for Fig. 5. Figure S1. Quantification of western blot analysis of nuclear respiratory factor 2 (NRF-2) in TA. Figure S2. Quantification of western blot analysis of nuclear respiratory factor 2 (NRF-2) in SOL. Figure S3. Glycolytic (SDH−) and oxidative (SDH+) myofiber count proportions across EDL and the effects of sex, HSAAR genotype, or endurance exercise on their distribution. [file 13293_2022_471_MOESM1_ESM.docx]

**Myocytic androgen receptor overexpression does not affect sex differences in adaptation to chronic endurance exercise**

**Sabrina T. Barsky, D. Ashley Monks***

**Additional Data**

**Table S1: Supplemental Statistics for exclusion criteria of exercising females. Endpoint 9-week DXA measurements were compared using non-paired, two-sided t-tests between conforming (CF) and non-conforming exercising females (nCF) to identify any effects of running style on body composition (nCF: *n*=10, CF: *n*=14)**

* denotes statistical significance

| **Variable** | **nCF Mean ± SEM** | **CF Mean ± SEM** | **T-statistic** | **P-value** |
| --- | --- | --- | --- | --- |
| Uncorrected Fat body mass (g) | 4.78 ± 1.08 | 6.51 ± 0.64 | -1.380 | 0.188 |
| Fat body mass % | 2.37 ± 0.50 | 2.91 ± 0.26 | -0.948 | 0.360 |
| Uncorrected Lean body mass (g) | 184.92 ± 4.19 | 208.02 ± 2.76 | -4.604 | 0.000278*** |
| Lean body mass % | 94.04 ± 0.47 | 93.56 ± 0.25 | 0.918 | 0.374 |
| Total body mass (g) | 196.79 ± 5.11 | 222.38 ± 3.11 | -4.275 | 0.000627*** |
| Bone mineral content (g) | 7.08 ± 0.16 | 7.87 ± 0.13 | -3.855 | 0.00104** |
| Bone mineral density (g/m^2^) | 0.153 ± 0.002 | 0.158 ± 0.002 | -2.121 | 0.0455* |

**Table S2: Supplemental Statistics for Figure 2. Type III Analysis of Variance With Satterthwaite’s Method (Linear Mixed Effects, LMER) of Sex (Between subjects, 2 levels, Male and Female), HSAAR Genotype (Between subjects, 2 levels, Tg and WT) and Physical Activity (Between subjects, 2 levels, Exercise and Sedentary) as main factors, and experimental block as the random factor**

* denotes statistical significance

Main effect of Sex

| **Variable** | **F-value** (*df* = 1,81) | **P-value** |
| --- | --- | --- |
| Uncorrected Fat body mass | 73.8696 | 1.02E-12*** |
| Fat body mass % | 21.0913 | 0.00001766*** |
| Uncorrected Lean body mass | 1020.4639 | < 2.00E-16*** |
| Lean body mass % | 12.5151 | 0.0007033*** |
| Total body mass | 918.2039 | < 2.20E-16*** |
| Bone mineral content | 481.8959 | < 2.20E-16*** |
| Bone mineral density | 52.7588 | 2.99E-10*** |

Main effect of HSAAR Genotype

| **Variable** | **F-value** (*df* = 1,81) | **P-value** |
| --- | --- | --- |
| Uncorrected Fat body mass | 2.549 | 0.1146 |
| Fat body mass % | 2.9882 | 0.087984 |
| Uncorrected Lean body mass | 5.5117 | 0.02149* |
| Lean body mass % | 3.1501 | 0.07998 |
| Total body mass | 1.8704 | 0.175471 |
| Bone mineral content | 1.5031 | 0.223999 |
| Bone mineral density | 0.7137 | 0.4009 |

Main effect of Physical Activity

| **Variable** | **F-value** (*df* = 1,81) | **P-value** |
| --- | --- | --- |
| Uncorrected Fat body mass | 54.8393 | 2.02E-10*** |
| Fat body mass % | 59.1402 | 5.92E-11*** |
| Uncorrected Lean body mass | 0.2848 | 0.59512 |
| Lean body mass % | 61.013 | 3.55E-11*** |
| Total body mass | 5.2631 | 0.0247114* |
| Bone mineral content | 2.7214 | 0.1033726 |
| Bone mineral density | 0.2399 | 0.6258 |

Interactive effects of Sex and HSAAR Genotype

| **Variable** | **F-value** (*df* = 1,81) | **P-value** |
| --- | --- | --- |
| Uncorrected Fat body mass | 1.4696 | 0.2292 |
| Fat body mass % | 1.0842 | 0.301083 |
| Uncorrected Lean body mass | 0.8403 | 0.36222 |
| Lean body mass % | 1.1998 | 0.2768517 |
| Total body mass | 0.0539 | 0.817112 |
| Bone mineral content | 0.1228 | 0.7270149 |
| Bone mineral density | 0.3493 | 0.5564 |

Interactive effects of Sex and Physical Activity

| **Variable** | **F-value** (*df* = 1,81) | **P-value** |
| --- | --- | --- |
| Uncorrected Fat body mass | 20.5183 | 0.00002284*** |
| Fat body mass % | 7.1315 | 0.009347** |
| Uncorrected Lean body mass | 5.7123 | 0.01932* |
| Lean body mass % | 7.6341 | 0.0072591** |
| Total body mass | 13.7874 | 0.0003972*** |
| Bone mineral content | 15.7453 | 0.0001679*** |
| Bone mineral density | 1.5976 | 0.2102 |

Interactive effects of HSAAR Genotype and Physical Activity

| **Variable** | **F-value** (*df* = 1,81) | **P-value** |
| --- | --- | --- |
| Uncorrected Fat body mass | 1.7769 | 0.1867 |
| Fat body mass % | 2.8828 | 0.093827 |
| Uncorrected Lean body mass | 1.6508 | 0.20275 |
| Lean body mass % | 2.5824 | 0.1124176 |
| Total body mass | 0.4288 | 0.5146232 |
| Bone mineral content | 3.3217 | 0.0724763 |
| Bone mineral density | 0.7472 | 0.3902 |

Interactive effects of Sex, HSAAR Genotype, and Physical Activity

| **Variable** | **F-value** (*df* = 1,81) | **P-value** |
| --- | --- | --- |
| Uncorrected Fat body mass | 0.2005 | 0.6556 |
| Fat body mass % | 0.0154 | 0.901551 |
| Uncorrected Lean body mass | 0.3986 | 0.52968 |
| Lean body mass % | 0.0058 | 0.9395084 |
| Total body mass | 0.1282 | 0.7212922 |
| Bone mineral content | 1.0818 | 0.3016026 |
| Bone mineral density | 0.0864 | 0.7696 |

**Table S3: Supplemental Statistics for Figure 2: Posthoc analysis using Two Sample T-tests on significant two-way effects indicated by LMER ANOVA. Unprotected P-values from multiple comparisons were corrected using Bonferroni correction. Adjusted P-values were compared to alpha.**

* denotes statistical significance

LMER denoted significant interactive effect of Sex and Physical Activity

| **Variable** | **Hypothesis** | **t-value** | **Corrected P-value** |
| --- | --- | --- | --- |
| Uncorrected Fat body mass | ME>FE | 7.2175 | 6.44E-08*** |
|  | FS>FE | 3.8679 | 1.51E-03** |
|  | MS>FE | 9.5699 | 3.97E-09*** |
|  | FS<ME | -1.6111 | 3.44E-01 |
|  | MS>ME | 6.9732 | 6.92E-07*** |
|  | MS>FS | 7.4731 | 9.71E-08*** |
| Fat body mass % | ME>FE | 3.0931 | 1.29E-02* |
|  | FS>FE | 4.2126 | 6.07E-04*** |
|  | MS>FE | 8.4172 | 1.43E-08*** |
|  | FS>ME | 2.6123 | 4.20E-02* |
|  | MS>ME | 7.1699 | 3.90E-07*** |
|  | MS>FS | 4.1375 | 4.90E-04*** |
| Uncorrected Lean body mass | ME>FE | 22.5600 | 1.19E-20*** |
|  | FS<FE | -3.2622 | 7.50E-03** |
|  | MS>FE | 23.2563 | 7.55E-21*** |
|  | FS<ME | -24.3902 | 4.69E-23*** |
|  | MS<ME | 1.0638 | 1.00E+00 |
|  | MS>FS | 25.0367 | 4.21E-23*** |
| Lean body mass % | ME<FE | -1.3038 | 6.05E-01 |
|  | FS<FE | -4.2987 | 4.85E-04*** |
|  | MS<FE | -7.7381 | 8.12E-08*** |
|  | FS<ME | -3.6213 | 3.16E-03** |
|  | MS<ME | -7.2112 | 3.22E-07*** |
|  | MS<FS | -3.5707 | 2.73E-03** |
| Total body mass | ME>FE | 21.6788 | 3.86E-20*** |
|  | FS>FE | -1.7955 | 1.00E+00 |
|  | MS>FE | 24.2478 | 6.09E-21*** |
|  | FS<ME | -22.7443 | 1.08E-21*** |
|  | MS>ME | 3.6704 | 1.98E-03** |
|  | MS>FS | 25.2192 | 3.80E-22*** |
| Bone mineral content | ME>FE | 12.3657 | 9.22E-14*** |
|  | FS>FE | -2.4703 | 1.00E+00 |
|  | MS>FE | 16.5925 | 1.27E-17*** |
|  | FS<ME | -15.4704 | 1.65E-15*** |
|  | MS>ME | 3.2772 | 6.16E-03** |
|  | MS>FS | 20.3161 | 4.50E-19*** |

**Figure S1:** Quantification of western blot analysis of nuclear respiratory factor 2 (NRF-2) in TA. Indicated groups: males (*yellow*), females (*green*), sedentary (*grey*), aerobic exercise (*red*), wild-type (*wt*) (blank fill), and HSAAR transgenic (*Tg*) (hatch fill). Data expressed in relative density (R.D.). Data are presented as means ± s.e.m and analyzed using three-way ANOVA, *n* = 5.

**Table S4: Supplemental Statistics for Figure 1A&3A-D: Analysis of Variance of Sex (Between subjects, 2 levels, Male and Female), HSAAR Genotype (Between subjects, 2 levels, Tg and WT) and Physical Activity (Between subjects, 2 levels, Exercise and Sedentary) as main factors in tibialis anterior (TA)**

* denotes statistical significance

Main effect of Sex

| **Protein (Tibialis Anterior)** | **F-value** (*df* = 1,37) | **P-value** |
| --- | --- | --- |
| MHC2a, myosin heavy chain 2a isoform | 36.45 | 3.11E-06*** |
| MHC1, myosin heavy chain 1 isoform | 6.432 | 0.0163* |
| PGC1a, peroxisome proliferator-activated receptor gamma coactivator 1 alpha | 3.719 | 0.0627 |
| TFAM, mitochondrial transcription factor A | 0.001 | 0.972 |
| NRF2, nuclear respiratory factor 2 | 0.929 | 0.342 |
| AR, androgen receptor | 17.363 | 0.000219*** |

Main effect of HSAAR Genotype

| **Protein (Tibialis Anterior)** | **F-value** (*df* = 1,37) | **P-value** |
| --- | --- | --- |
| MHC2a, myosin heavy chain 2a isoform | 1.236 | 0.277 |
| MHC1, myosin heavy chain 1 isoform | 0.301 | 0.587 |
| PGC1a, peroxisome proliferator-activated receptor gamma coactivator 1 alpha | 0.016 | 0.9007 |
| TFAM, mitochondrial transcription factor A | 0.177 | 0.677 |
| NRF2, nuclear respiratory factor 2 | 0.255 | 0.617 |
| AR, androgen receptor | 131.437 | 7.23E-13*** |

Main effect of Physical Activity

| **Protein (Tibialis Anterior)** | **F-value** (*df* = 1,37) | **P-value** |
| --- | --- | --- |
| MHC2a, myosin heavy chain 2a isoform | 1.895 | 0.181 |
| MHC1, myosin heavy chain 1 isoform | 1.342 | 0.2552 |
| PGC1a, peroxisome proliferator-activated receptor gamma coactivator 1 alpha | 0.028 | 0.869 |
| TFAM, mitochondrial transcription factor A | 0.738 | 0.397 |
| NRF2, nuclear respiratory factor 2 | 0.187 | 0.668 |
| AR, androgen receptor | 4.676 | 0.038173* |

Interactive effects of Sex and HSAAR Genotype

| **Protein (Tibialis Anterior)** | **F-value** (*df* = 1,37) | **P-value** |
| --- | --- | --- |
| MHC2a, myosin heavy chain 2a isoform | 0.005 | 0.942 |
| MHC1, myosin heavy chain 1 isoform | 0.79 | 0.3808 |
| PGC1a, peroxisome proliferator-activated receptor gamma coactivator 1 alpha | 0.061 | 0.8062 |
| TFAM, mitochondrial transcription factor A | 0 | 0.995 |
| NRF2, nuclear respiratory factor 2 | 0.489 | 0.489 |
| AR, androgen receptor | 11.452 | 0.001903** |

Interactive effects of Sex and Physical Activity

| **Protein (Tibialis Anterior)** | **F-value** (*df* = 1,37) | **P-value** |
| --- | --- | --- |
| MHC2a, myosin heavy chain 2a isoform | 0.356 | 0.556 |
| MHC1, myosin heavy chain 1 isoform | 0.117 | 0.7347 |
| PGC1a, peroxisome proliferator-activated receptor gamma coactivator 1 alpha | 0.198 | 0.6589 |
| TFAM, mitochondrial transcription factor A | 0.109 | 0.744 |
| NRF2, nuclear respiratory factor 2 | 0.792 | 0.38 |
| AR, androgen receptor | 0.928 | 0.342584 |

Interactive effects of HSAAR Genotype and Physical Activity

| **Protein (Tibialis Anterior)** | **F-value** (*df* = 1,37) | **P-value** |
| --- | --- | --- |
| MHC2a, myosin heavy chain 2a isoform | 0.006 | 0.941 |
| MHC1, myosin heavy chain 1 isoform | 0.455 | 0.5047 |
| PGC1a, peroxisome proliferator-activated receptor gamma coactivator 1 alpha | 0.291 | 0.5934 |
| TFAM, mitochondrial transcription factor A | 0.096 | 0.759 |
| NRF2, nuclear respiratory factor 2 | 1.085 | 0.305 |
| AR, androgen receptor | 1.565 | 0.220016 |

Interactive effects of Sex, HSAAR Genotype, and Physical Activity

| **Protein (Tibialis Anterior)** | **F-value** (*df* = 1,37) | **P-value** |
| --- | --- | --- |
| MHC2a, myosin heavy chain 2a isoform | 0.011 | 0.918 |
| MHC1, myosin heavy chain 1 isoform | 0.222 | 0.6405 |
| PGC1a, peroxisome proliferator-activated receptor gamma coactivator 1 alpha | 6.089 | 0.0191* |
| TFAM, mitochondrial transcription factor A | 0.105 | 0.748 |
| NRF2, nuclear respiratory factor 2 | 0.017 | 0.898 |
| AR, androgen receptor | 0.299 | 0.58821 |

**Table S5: Supplemental Statistics for Figure 1A&3C: Post-hoc Tukey used for multiple comparisons when indicated by ANOVA significance. P-values are compared to alpha.**

* denotes statistical significance

ANOVA denoted significant interactive effect of Sex and HSAAR Genotype

| **Protein (Tibialis Anterior)** | **Hypothesis** | **P-value** |
| --- | --- | --- |
| AR, androgen receptor | MT>FT | 0.0000423*** |
|  | FW<FT | 0.0000143*** |
|  | MW<FT | 0.0000709*** |
|  | FW<MT | 0*** |
|  | MW<MT | 0*** |
|  | MW>FW | 0.9449005 |

ANOVA denoted significant interactive effect of Sex, Physical Activity, and HSAAR Genotype

| **Protein (Tibialis Anterior)** | **Hypothesis** | **P-value** |
| --- | --- | --- |
| PGC1a, peroxisome proliferator-activated receptor gamma coactivator 1 alpha | MTE<FTE | 1 |
|  | FEW<FTE | 0.966982 |
|  | MWE>FTE | 0.791458 |
|  | FTS<FTE | 0.990326 |
|  | MTS>FTE | 0.966621 |
|  | FWS>FTE | 1 |
|  | MWS<FTE | 0.999989 |
|  | FEW<MTE | 0.988078 |
|  | MWE>MTE | 0.693271 |
|  | FTS<MTE | 0.997644 |
|  | MTS>MTE | 0.926753 |
|  | FWS>MTE | 0.999996 |
|  | MWS<MTE | 1 |
|  | MWE>FWE | 0.213779 |
|  | FTS>FWE | 0.999999 |
|  | MTS>FWE | 0.465254 |
|  | FWS>FWE | 0.951274 |
|  | MWS>FWE | 0.995137 |
|  | FTS<MWE | 0.302252 |
|  | MTS<MWE | 0.999622 |
|  | FWS<MWE | 0.831528 |
|  | MWS<MWE | 0.618646 |
|  | MTS>FTS | 0.591671 |
|  | FWS>FTS | 0.98358 |
|  | MWS>FTS | 0.999351 |
|  | FWS<MTS | 0.978429 |
|  | MWS<MTS | 0.885712 |
|  | MWS<FWS | 0.999945 |

**Figure S2:** Quantification of western blot analysis of nuclear respiratory factor 2 (NRF-2) in SOL. Indicated groups: males (*yellow*), females (*green*), sedentary (*grey*), aerobic exercise (*red*), wild-type (*wt*) (blank fill), and HSAAR transgenic (*Tg*) (hatch fill). Data expressed in relative density (R.D.). Data are presented as means ± s.e.m and analyzed using three-way ANOVA, *n* = 4.

**Table S6: Supplemental Statistics for Figure 3E-H: Analysis of Variance of Sex (Between subjects, 2 levels, Male and Female), HSAAR Genotype (Between subjects, 2 levels, Tg and WT) and Physical Activity (Between subjects, 2 levels, Exercise and Sedentary) as main factors in soleus (SOL)**

* denotes statistical significance

Main effect of Sex

| **Protein (Soleus)** | **F-value** (*df* = 1,29) | **P-value** |
| --- | --- | --- |
| MHC2a, myosin heavy chain 2a isoform | 1.072 | 0.3108 |
| MHC1, myosin heavy chain 1 isoform | 0.935 | 0.343 |
| PGC1a, peroxisome proliferator-activated receptor gamma coactivator 1 alpha | 4.024 | 0.0563 |
| TFAM, mitochondrial transcription factor A | 4.73 | 0.0397* |
| NRF2, nuclear respiratory factor 2 | 0.524 | 0.476 |

Main effect of HSAAR Genotype

| **Protein (Soleus)** | **F-value** (*df* = 1,29) | **P-value** |
| --- | --- | --- |
| MHC2a, myosin heavy chain 2a isoform | 5.643 | 0.0259* |
| MHC1, myosin heavy chain 1 isoform | 0.003 | 0.956 |
| PGC1a, peroxisome proliferator-activated receptor gamma coactivator 1 alpha | 0.169 | 0.685 |
| TFAM, mitochondrial transcription factor A | 0.262 | 0.6133 |
| NRF2, nuclear respiratory factor 2 | 0.122 | 0.73 |

Main effect of Physical Activity

| **Protein (Soleus)** | **F-value** (*df* = 1,29) | **P-value** |
| --- | --- | --- |
| MHC2a, myosin heavy chain 2a isoform | 1.412 | 0.2463 |
| MHC1, myosin heavy chain 1 isoform | 0.903 | 0.351 |
| PGC1a, peroxisome proliferator-activated receptor gamma coactivator 1 alpha | 0.038 | 0.8475 |
| TFAM, mitochondrial transcription factor A | 0.079 | 0.7808 |
| NRF2, nuclear respiratory factor 2 | 0.032 | 0.859 |

Interactive effects of Sex and HSAAR Genotype

| **Protein (Soleus)** | **F-value** (*df* = 1,29) | **P-value** |
| --- | --- | --- |
| MHC2a, myosin heavy chain 2a isoform | 1.944 | 0.176 |
| MHC1, myosin heavy chain 1 isoform | 0.023 | 0.88 |
| PGC1a, peroxisome proliferator-activated receptor gamma coactivator 1 alpha | 0.314 | 0.5802 |
| TFAM, mitochondrial transcription factor A | 0.079 | 0.7811 |
| NRF2, nuclear respiratory factor 2 | 0.043 | 0.838 |

Interactive effects of Sex and Physical Activity

| **Protein (Soleus)** | **F-value** (*df* = 1,29) | **P-value** |
| --- | --- | --- |
| MHC2a, myosin heavy chain 2a isoform | 0.08 | 0.7797 |
| MHC1, myosin heavy chain 1 isoform | 2.098 | 0.16 |
| PGC1a, peroxisome proliferator-activated receptor gamma coactivator 1 alpha | 2.194 | 0.1516 |
| TFAM, mitochondrial transcription factor A | 0.005 | 0.9442 |
| NRF2, nuclear respiratory factor 2 | 0.008 | 0.931 |

Interactive effects of HSAAR Genotype and Physical Activity

| **Protein (Soleus)** | **F-value** (*df* = 1,29) | **P-value** |
| --- | --- | --- |
| MHC2a, myosin heavy chain 2a isoform | 0.651 | 0.4277 |
| MHC1, myosin heavy chain 1 isoform | 0.019 | 0.892 |
| PGC1a, peroxisome proliferator-activated receptor gamma coactivator 1 alpha | 0.005 | 0.9454 |
| TFAM, mitochondrial transcription factor A | 0.098 | 0.7567 |
| NRF2, nuclear respiratory factor 2 | 0.128 | 0.723 |

Interactive effects of Sex, HSAAR Genotype, and Physical Activity

| **Protein (Soleus)** | **F-value** (*df* = 1,29) | **P-value** |
| --- | --- | --- |
| MHC2a, myosin heavy chain 2a isoform | 0.019 | 0.8917 |
| MHC1, myosin heavy chain 1 isoform | 1.356 | 0.256 |
| PGC1a, peroxisome proliferator-activated receptor gamma coactivator 1 alpha | 0.415 | 0.5254 |
| TFAM, mitochondrial transcription factor A | 0.049 | 0.8267 |
| NRF2, nuclear respiratory factor 2 | 0.01 | 0.921 |

**Table S7: Supplemental Statistics for Figure 4B,C: Analysis of Variance of Sex (Between subjects, 2 levels, Male and Female), HSAAR Genotype (Between subjects, 2 levels, Tg and WT) and Physical Activity (Between subjects, 2 levels, Exercise and Sedentary) as main factors in minor ellipse of hemotoxylin and eosin (H&E) matched succinate dehydrogenase stained (SDH+) or unstained (SDH-) myofibers of extensor digitorum longus (EDL)**

* denotes statistical significance

Main effect of Sex

| **Myofiber** | **F-value** (*df* = 1,45) | **P-value** |
| --- | --- | --- |
| SDH+ | 16.846 | 0.000194*** |
| SDH- | 40.03 | 1.64E-07*** |

Main effect of HSAAR Genotype

| **Myofiber** | **F-value** (*df* = 1,45) | **P-value** |
| --- | --- | --- |
| SDH+ | 0.864 | 0.35818 |
| SDH- | 7.837 | 0.00784** |

Main effect of Physical Activity

| **Myofiber** | **F-value** (*df* = 1,45) | **P-value** |
| --- | --- | --- |
| SDH+ | 0.276 | 0.602395 |
| SDH- | 0.184 | 0.67001 |

Interactive effects of Sex and HSAAR Genotype

| **Myofiber** | **F-value** (*df* = 1,45) | **P-value** |
| --- | --- | --- |
| SDH+ | 5.493 | 0.024148* |
| SDH- | 0.049 | 0.82521 |

Interactive effects of Sex and Physical Activity

| **Myofiber** | **F-value** (*df* = 1,45) | **P-value** |
| --- | --- | --- |
| SDH+ | 1.237 | 0.272601 |
| SDH- | 1.178 | 0.28417 |

Interactive effects of HSAAR Genotype and Physical Activity

| **Myofiber** | **F-value** (*df* = 1,45) | **P-value** |
| --- | --- | --- |
| SDH+ | 0.775 | 0.383838 |
| SDH- | 2.387 | 0.13026 |

Interactive effects of Sex, HSAAR Genotype, and Physical Activity

| **Myofiber** | **F-value** (*df* = 1,45) | **P-value** |
| --- | --- | --- |
| SDH+ | 0.689 | 0.411463 |
| SDH- | 0.068 | 0.79586 |

**Table S8: Supplemental Statistics for Figure 4B: Post-hoc Tukey used for multiple comparisons when indicated by ANOVA significance. P-values are compared to alpha.**

* denotes statistical significance

ANOVA denoted significant interactive effect of Sex and HSAAR Genotype

| **Myofiber** | **Hypothesis** | **P-value** |
| --- | --- | --- |
| SDH+ | MT>FT | 0.6025281 |
|  | FW<FT | 0.7502609 |
|  | MW>FT | 0.0051862** |
|  | FW<MT | 0.1286837 |
|  | MW>MT | 0.1117002 |
|  | MW>FW | 0.0002694*** |

**Figure S3:** Glycolytic (SDH-) and oxidative (SDH+) myofiber count proportions across EDL and the effects of sex, HSAAR genotype, or endurance exercise on their distribution. Distribution of glycolytic (a,c) and oxidative (b,d) myofiber count proportion across each 40x magnified photomicrographs. Data presented as frequency distribution and collapsed by sex (a), HSAAR genotype (b), and exercise (c-d). (a) *n* = 24 per sex (collapses n=6 transgene-exercise, n=6 wildtype-exercise, n=6 transgene-sedentary, n=6 wildtype-sedentary). (b) *n* = 24 per genotype (collapses n=6 female-exercise, n=6 male-exercise, n=6 female-sedentary, n=6 male-sedentary). (c,d) *n* = 24 per activity treatment (collapses n=6 female-transgene, n=6 male-transgene, n=6 female-wildtype, n=6 male-wildtype). All data analyzed using three-way ANOVA. Main effect by sex: **P* < 0.05.

**Table S9: Supplemental Statistics for Figure 4D,E: Analysis of Variance of Sex (Between subjects, 2 levels, Male and Female), HSAAR Genotype (Between subjects, 2 levels, Tg and WT) and Physical Activity (Between subjects, 2 levels, Exercise and Sedentary) as main factors in glycolytic (SDH-) and oxidative (SDH+) myofiber count proportions in extensor digitorum longus (EDL)**

* denotes statistical significance

Main effect of Sex

| **Myofiber** | **F-value** (*df* = 1,45) | **P-value** |
| --- | --- | --- |
| SDH+ | 4.205 | 0.0469* |
| SDH- | 4.205 | 0.0469* |

Main effect of HSAAR Genotype

| **Myofiber** | **F-value** (*df* = 1,45) | **P-value** |
| --- | --- | --- |
| SDH+ | 0.62 | 0.4356 |
| SDH- | 0.62 | 0.4356 |

Main effect of Physical Activity

| **Myofiber** | **F-value** (*df* = 1,45) | **P-value** |
| --- | --- | --- |
| SDH+ | 2.017 | 0.1633 |
| SDH- | 2.017 | 0.1633 |

Interactive effects of Sex and HSAAR Genotype

| **Myofiber** | **F-value** (*df* = 1,45) | **P-value** |
| --- | --- | --- |
| SDH+ | 0.002 | 0.9638 |
| SDH- | 0.002 | 0.9638 |

Interactive effects of Sex and Physical Activity

| **Myofiber** | **F-value** (*df* = 1,45) | **P-value** |
| --- | --- | --- |
| SDH+ | 1.516 | 0.2254 |
| SDH- | 1.516 | 0.2254 |

Interactive effects of HSAAR Genotype and Physical Activity

| **Myofiber** | **F-value** (*df* = 1,45) | **P-value** |
| --- | --- | --- |
| SDH+ | 2.554 | 0.1179 |
| SDH- | 2.554 | 0.1179 |

Interactive effects of Sex, HSAAR Genotype, and Physical Activity

| **Myofiber** | **F-value** (*df* = 1,45) | **P-value** |
| --- | --- | --- |
| SDH+ | 3.395 | 0.0728 |
| SDH- | 3.395 | 0.0728 |

**Table S10: Supplemental Statistics for Figure 5: Pearson correlation output between exercise (*n*=16) and sedentary (*n*=16) intramuscular AR expression and fat body mass, lean body mass, glycolytic minor ellipse, and glycolytic myofiber proportion**

* denotes statistical significance

| **Variable** | **Factor** | **Correlation** | **T-value** | **P-value** |
| --- | --- | --- | --- | --- |
| AR and Fat body mass | Exercise | 0.106 | 0.427 | 0.6754 |
|  | Sedentary | -0.0204 | -0.082 | 0.936 |
| AR and Lean body mass | Exercise | 0.223 | 0.916 | 0.3733 |
|  | Sedentary | 0.375 | 1.616 | 0.1255 |
| AR and SDH- Minor Ellipse | Exercise | 0.564 | 2.736 | 0.01466* |
|  | Sedentary | 0.316 | 1.332 | 0.2017 |
| AR and SDH- Proportion | Exercise | 0.465 | 2.103 | 0.05163 |
|  | Sedentary | 0.201 | 0.823 | 0.4227 |
